# Taxonomy of *Rhizobiaceae* revisited: proposal of a new framework for genus delimitation

**DOI:** 10.1099/ijsem.0.005243

**Published:** 2022-03-03

**Authors:** Nemanja Kuzmanović, Camilla Fagorzi, Alessio Mengoni, Florent Lassalle, George C. diCenzo

**Affiliations:** ^1^​ Julius Kühn Institute, Federal Research Centre for Cultivated Plants (JKI), Institute for Plant Protection in Horticulture and Forests, Braunschweig, Germany; ^2^​ Department of Biology, University of Florence, Florence, Italy; ^3^​ Parasites and Microbes, Wellcome Sanger Institute, Wellcome Genome Campus, Hinxton, Cambridgeshire, UK; ^4^​ Department of Biology, Queen’s University, Kingston, Ontario, Canada

**Keywords:** *Rhizobiaceae*, *Xaviernesmea*, *Ensifer*, *Sinorhizobium*, *Rhizobium*, genus boundaries

## Abstract

The alphaproteobacterial family *Rhizobiaceae* is highly diverse, with 168 species with validly published names classified into 17 genera with validly published names. Most named genera in this family are delineated based on genomic relatedness and phylogenetic relationships, but some historically named genera show inconsistent distribution and phylogenetic breadth. The most problematic is *Rhizobium *, which is notorious for being highly paraphyletic, as most newly described species in the family are assigned to this genus without consideration of their proximity to existing genera, or the need to create novel genera. Moreover, many *Rhizobiaceae* genera lack synapomorphic traits that would give them biological and ecological significance. We propose a common framework for genus delimitation within the family *Rhizobiaceae *, wherein genera are defined as monophyletic groups in a core-genome gene phylogeny, that are separated from related species using a pairwise core-proteome average amino acid identity (cpAAI) threshold of approximately 86 %. We further propose that additional genomic or phenotypic evidence can justify division of species into separate genera even if they share greater than 86 % cpAAI. Applying this framework, we propose to reclassify *Rhizobium rhizosphaerae* and *Rhizobium oryzae* into *Xaviernesmea* gen. nov. Data is also provided to support the formation of *Peteryoungia aggregata* comb. nov., *Endobacterium yantingense* comb. nov., *Neorhizobium petrolearium* comb. nov., *Pararhizobium arenae* comb. nov., *Pseudorhizobium tarimense* comb. nov. and *Mycoplana azooxidifex* comb. nov. Lastly, we present arguments that the unification of the genera *Ensifer* and *Sinorhizobium* in Opinion 84 of the Judicial Commission is no longer justified by current genomic and phenotypic data. Despite pairwise cpAAI values for all *Ensifer* species and all *Sinorhizobium* species being >86 %, additional genomic and phenotypic data suggest that they significantly differ in their biology and ecology. We therefore propose emended descriptions of *Ensifer* and *Sinorhizobium *, which we argue should be considered as separate genera.

## Data Summary

All genome sequences used in this work were previously published, and the assembly accessions are provided in Dataset S1 (available in the online version of this article). Twelve supplementary figures and three supplementary datasets are included in the online version of this article. All raw data (core-proteome average amino acid identity data, whole-proteome average amino acid identity data, average nucleotide identity data, percentage of conserved proteins, and a Newick formatted phylogeny) used to generate the figures presented in this manuscript are available through Figshare at https://doi.org/10.6084/m9.figshare.17076455.v1 [1]. A pipeline to extract the 170 marker proteins used for phylogenetic reconstruction and in calculating core-proteome average amino acid identity values, is available through GitHub at github.com/flass/cpAAI_Rhizobiaceae.

## Introduction

The family *Rhizobiaceae* of the order *Alphaproteobacteria* was proposed in 1938 and has since undergone numerous, and at times contentious, taxonomic revisions [2, 3]. Currently, this family comprises the genera *Agrobacterium *, *Allorhizobium *, *Ciceribacter *, *Endobacterium *, *Ensifer* (syn. *Sinorhizobium *), *Gellertiella *, *Georhizobium *, *Hoeflea *, *Lentilitoribacter *, *Liberibacter *, *Martelella *, *Mycoplana *, ‘* Neopararhizobium *’, *Neorhizobium *, *Pararhizobium *, *Peteryoungia *, *Pseudorhizobium *, *Rhizobium* and *Shinella* (syn. *Crabtreella *; https://lpsn.dsmz.de/) [4]. The family *Rhizobiaceae* contains phenotypically diverse organisms, including N_2_-fixing legume symbionts (known as rhizobia), plant pathogens, bacterial predators, and other soil bacteria. The agricultural and ecological significance of the family *Rhizobiaceae* has prompted the isolation and whole genome sequencing of hundreds of strains at a rate outpacing taxonomic refinement of the family. As a result, some species and genera within the family are well known to be paraphyletic [5], while others that are monophyletic likely represent multiple species/genera [6]. In addition, most currently named genera have been delineated based on genomic relatedness – as per current taxonomic guidelines [7] – but lack synapomorphic traits that would give them biological and ecological significance [8].

To aid in the taxonomic classification of this family, here we propose a general framework for defining genera in the family *Rhizobiaceae *. This framework is based on a set of baseline genomic relatedness measures meant to serve as minimal thresholds for genus demarcation, while allowing for more closely related species to be divided into separate genera when supported by supplemental genomic and/or biological data. By applying this framework, we propose the formation of a new genus – *Xaviernesmea* – on the basis of the genomic relatedness measures, and provide support for the recently described genus *Peteryoungia *. In addition, despite genomic relatedness values that would not support genus demarcation based on the proposed baseline thresholds, we argue that current phylogenetic, genomic (e.g. pentanucleotide frequency) and biological (e.g. division by budding) data indicate that the genera *Ensifer* Casida *et al*. 1982 [9] and *Sinorhizobium* Chen 1988 [10] are not synonymous, meaning that the unification of the genera *Ensifer* and *Sinorhizobium* in Opinion 84 of the Judicial Commission is no longer justified.

## Methods

### Dataset

The analysis was performed on a dataset of 94 genomes of *Rhizobiaceae* strains, among which the majority were type strains of the corresponding species (Dataset S1). As an outgroup, we included the genomes of three *Mesorhizobium* strains, belonging to the related family *Phyllobacteriaceae *. Moreover, for calculation of some overall genome relatedness indices (OGRIs) to support additional taxonomic revisions, genomes of two *Pararhizobium* and two *Pseudorhizobium* strains were included (Dataset S1). To verify the authenticity of genomes used for taxonomic reclassifications proposed in this paper, we compared the reference 16S rRNA gene sequences (as well as housekeeping gene sequences in ambiguous cases) associated with the original species publication with the sequences retrieved from genome sequences (Dataset S1). Whole genome sequences generated to support new species description in original publications were considered as authentic (Dataset S1).

### Core-genome gene phylogeny

The core-genome phylogeny was obtained using the GET_HOMOLOGUES software package version 10032020 [11] and the GET_PHYLOMARKERS software package version 2.2.8_18Nov2018 [12], as described previously [13]. As a result, a set of 170 non-recombining single-copy core marker genes was selected, and a concatenation of their codon-based alignments was used as input for IQ-TREE ModelFinder, with which a search for the best sequence evolution model was conducted. The model ‘GTR+F+ASC+R8’ was selected based on a Bayesian information criterion. The maximum-likelihood (ML) core genome phylogeny was inferred under this model using IQ-TREE [14], with branch supports assessed with approximate Bayes test (-abayes) and ultrafast bootstrap with 1000 replicates (-bb 1000).

### Overall genome relatedness indices calculations

Whole-proteome average amino-acid identity (wpAAI; usually simply known as AAI) was computed using the CompareM software (github.com/dparks1134/CompareM) using the aai_wf command with default parameters, i.e., ortholog identification with DIAMOND [15], e-value <1e-3, percent identity >30 %, and alignment length >70 % the length of the protein.

Core-proteome average amino-acid identity (cpAAI) was computed as the proportion of substitutions in pairwise comparisons of sequences from the 170 non-recombining, single-copy core marker genes identified using GET_PHYLOMARKERS [12], using a custom R script that notably relied on the dist.aa() function from the ‘ape’ package [16].

Percentage of conserved proteins (POCP) was determined using publicly available code (github.com/hoelzer/pocp) and the ortholog identification thresholds defined by Qin *et al*. [17], namely, e-value <1e-5, percent identity >40 %, and alignment length >50 % the length of the protein. This pipeline involved the reannotation of genomes with Prodigal version 2.6.3 [18] and ortholog identification using the blast+ package, version 2.10.1 [19].

The average nucleotide identity (ANIb) comparisons were conducted using PyANI version 0.2.9, with scripts employing the blast+ algorithm to align the input sequences (https://github.com/widdowquinn/pyani). The digital DNA–DNA hybridization (dDDH) computations were performed with the Genome-to-Genome Distance Calculator (GGDC 2.1; https://ggdc.dsmz.de/distcalc2.php) using the recommended blast+ alignment and formula 2 (identities/HSP length) [20].

### 16S rRNA gene phylogeny

The RNA fasta files for the 157 *Sinorhizobium* or *Ensifer* strains analysed in our recent study [21] were downloaded from the National Centre for Biotechnology Information database, and all 16S rRNA gene sequences ≥1000 nt were extracted. The 16S rRNA gene sequences were aligned using mafft version 7.3.10 with the localpair option [22], and trimmed using trimAl version 1.4.rev22 with the automated1 option [23]. A ML phylogeny was prepared using raxmlHPC-HYBRID-AVX2 version 8.2.12 with the GTRCAT model [24]. The final phylogeny is the bootstrap best tree following 756 bootstrap replicates, as determined by the extended majority-rule consensus tree criterion.

### Code availability

To facilitate the adoption of our framework for genus demarcation in the family *Rhizobiaceae *, a custom pipeline was prepared to extract the 170 marker proteins from other genomes. The pipeline, together with the 170 marker proteins from the 97 strains analysed in the current study, is available at github.com/flass/cpAAI_Rhizobiaceae. Briefly, the pipeline will use tblastn [19] to identify genes encoding orthologs of all 170 marker proteins in the input genomes, which will then be extracted and translated. Each set of protein orthologs is then aligned with mafft [22] or Clustal Omega [25] to the pre-computed alignment of the orthologs from the 97 strains analysed here, and a concatenated alignment prepared. The output files can then be used for phylogenetic reconstruction or cpAAI calculations, with sample code for cpAAI calculations also provided.

## Results and discussion

### Overall genomic relatedness indices measurements in the family *Rhizobiaceae*

**Fig. 1.**
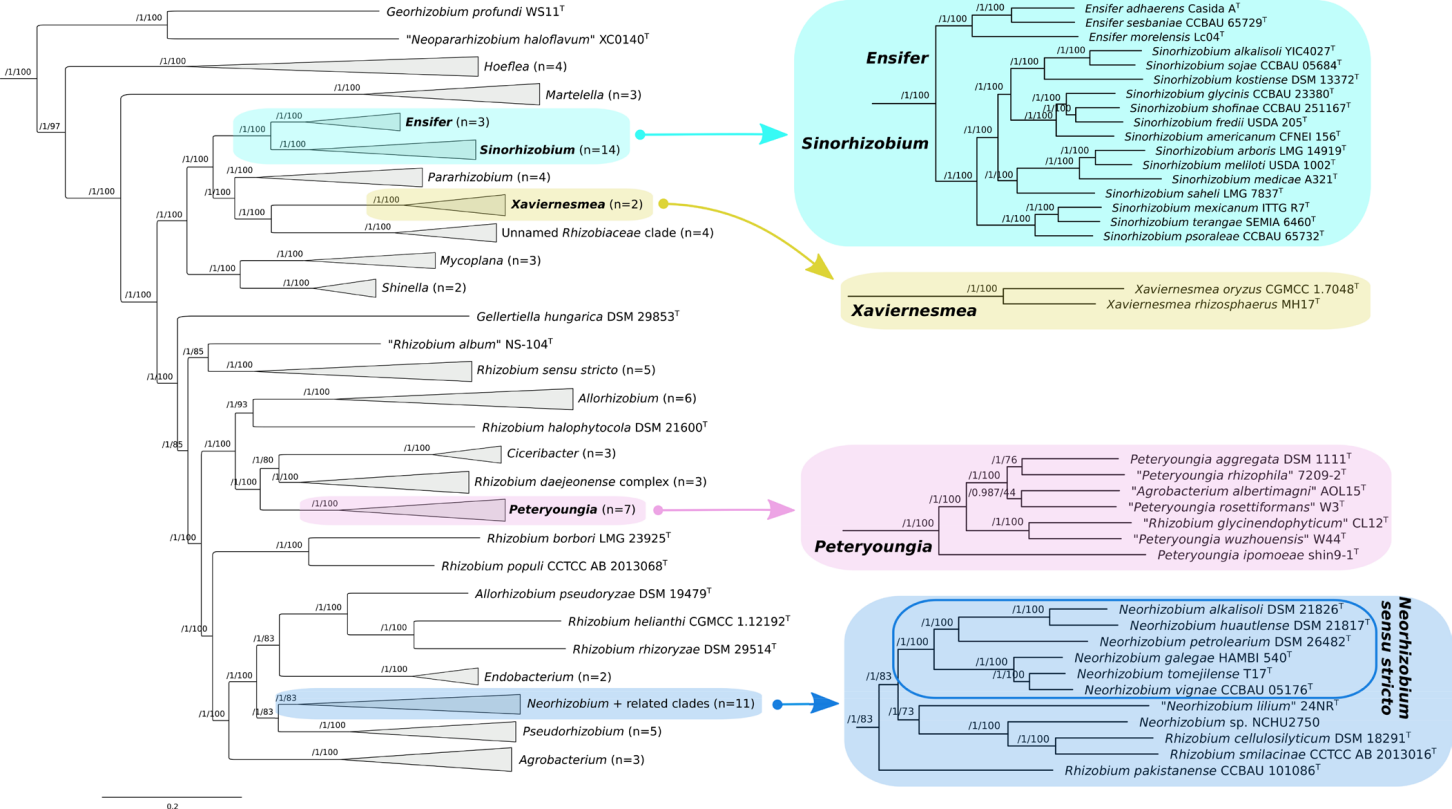
Maximum-likelihood core-genome phylogeny of the family *Rhizobiaceae *. A maximum-likelihood phylogeny 94 *Rhizobiaceae* strains is shown. The number of strains included in each collapsed clade is indicated. Clades of focus in the current study are expanded along the righthand side of the figure. The phylogeny is built from the concatenated alignments of 170 nonrecombinant loci using IQ-TREE [14]. The numbers on the nodes indicate the approximate Bayesian posterior probabilities support values (first value) and ultra-fast bootstrap values (second value). The tree was rooted using three *Mesorhizobium* spp. sequences as the outgroup. The scale bar represents the number of expected substitutions per site under the best-fitting GTR+F+ASC+R8 model. An expanded phylogeny is provided as Fig. S1.

To develop a framework for genus demarcation within the family *Rhizobiaceae *, we examined a selection of 94 genomes of *Rhizobiaceae* isolates, most of which are species type strains. *Liberibacter *, an obligate intra-cellular pathogen with a highly reduced genome, was excluded from our selection of organisms to avoid biassing the analysis by overly reducing the conserved gene set. We reasoned that good practices for genome sequence-based genus delineation should consider both phylogenetic relatedness of species based on a concatenated alignment of core-genome genes (Figs 1 and S1), and one or more OGRI measurement. We initially considered four OGRIs, calculated as described in the Methods: (i) average nucleotide identity (ANIb), (ii) whole-proteome average amino acid identity (wpAAI); (iii) core-proteome average amino acid identity (cpAAI) based on the proportion of substitutions between the concatenated translated sequences of the core marker gene set used for the core-genome phylogeny; and (iv) the percentage of conserved proteins (POCP) as defined by Qin *et al*. [17]. Digital DNA–DNA hybridization (dDDH; Dataset S3) was also performed for some strains to verify they represented distinct species; however, dDDH was not considered when defining genera.

**Fig. 2.**
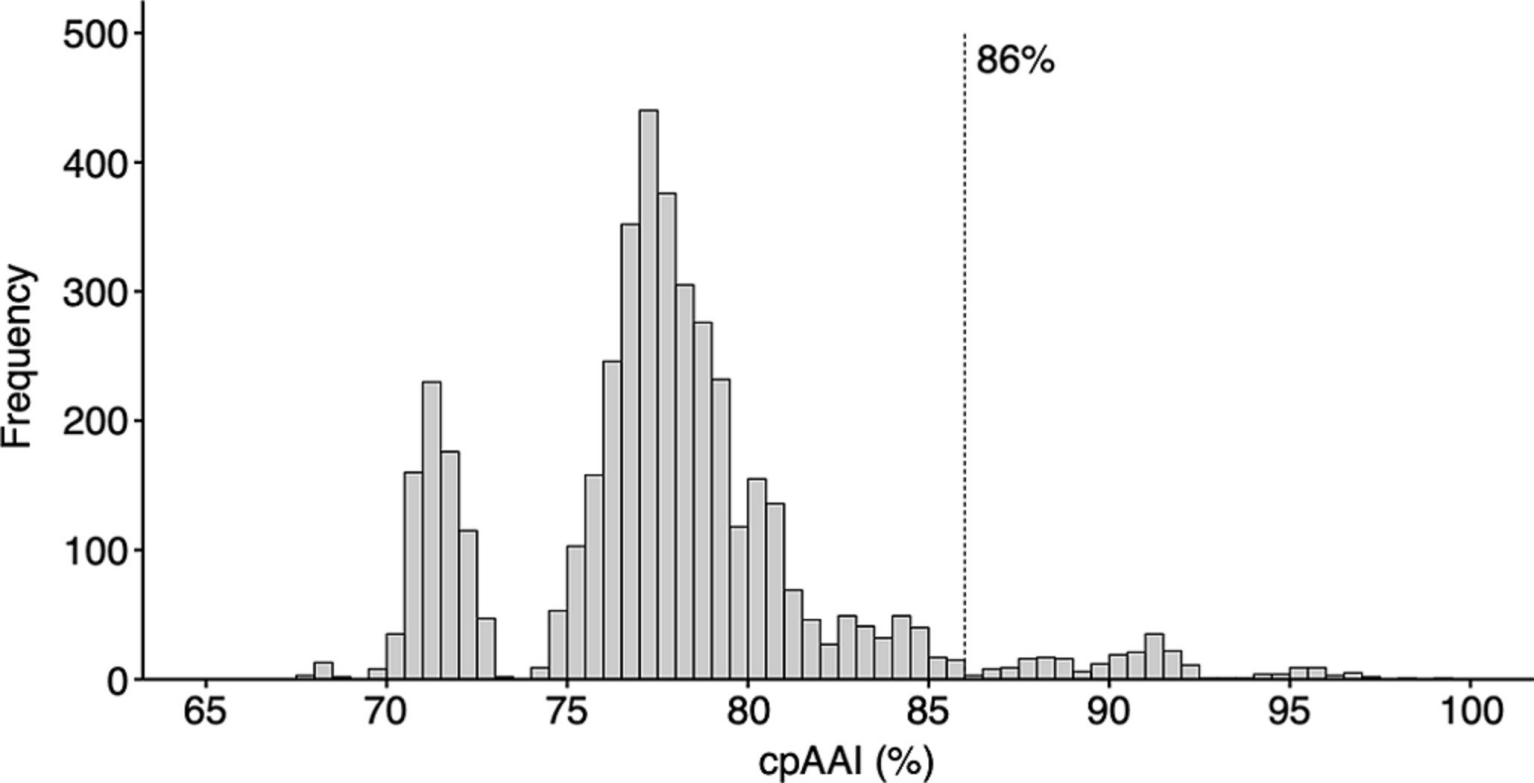
Distribution of core-proteome AAI (cpAAI) comparisons of the family *Rhizobiaceae *. Pairwise cpAAI values were calculated based on 170 nonrecombinant loci from the core-genome of 94 members of the family *Rhizobiaceae.* Results are summarized as a histogram with a bin width of 0.5 %.

On the assumption that genera are not artificial divisions of a continuum of species, but that they instead represent biologically meaningful differentiation of groups of species, we reasoned that an OGRI threshold for delimiting genera should correspond to a drop in the OGRI frequency distribution. We therefore plotted histograms of all pairwise comparisons to identify potential genera boundaries (Figs 2 and S2–S4). It was previously suggested that a 50 % POCP threshold is a good measure of genus boundaries in other families [17]. However, we found that 3885 out of the 4371 (89 %) pairwise comparisons in our *Rhizobiaceae* dataset gave a POCP value ≥50 %, with no clear breaks in the frequency distribution (Fig. S2). We therefore concluded that POCP is not a useful OGRI measurement for defining genera in the family *Rhizobiaceae *. Similar observations were also reported for some other taxa, such as members of the roseobacter group [26].

**Fig. 3.**
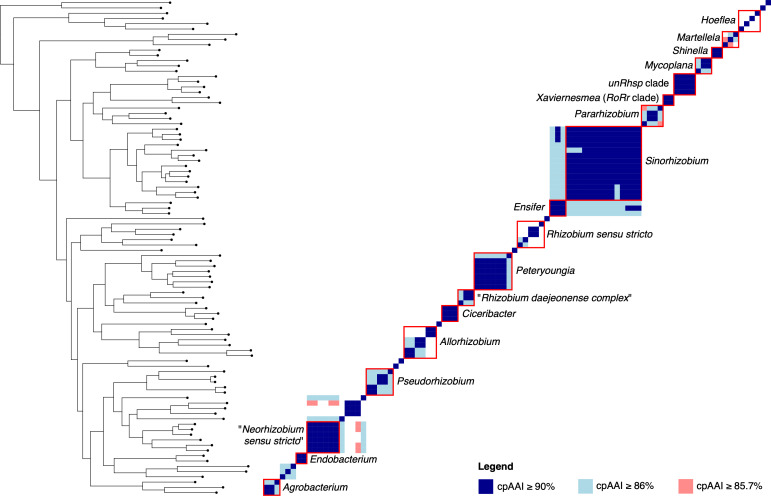
Core-proteome AAI (cpAAI) matrix of the family *Rhizobiaceae *. A matrix showing the pairwise cpAAI values for each pair of 94 members of the family *Rhizobiaceae *. Values were clustered using the core-genome gene phylogeny of Figs 1 and S1. Several named genera are indicated with red boxes, as indicated. A version of this matrix with a colour scheme representing the full range of cpAAI values is provided as Fig. S6.

cpAAI data was recently used to delineate genera among other bacterial families [26, 27] and stands as a promising metric for genus demarcation in the family *Rhizobiaceae* (Fig. 2). We observed a break in the frequency distribution at ~93 % to~94 %, but it was too stringent to use for genus demarcation as it would result in the majority of the 94 strains being classified into their own genera. Likewise, the break at ~73 % to~74 % was too lenient for genus delimitation as all strains would be grouped as a single genus, except for those belonging to the genera *Martelella *, *Hoeflea *, ‘* Neopararhizobium *’, and *Georhizobium *. Instead, the drop in the frequency distribution at 86–86.7 % (inclusive), within which only five of the 4371 pairwise comparisons fell, appeared to be a reasonable threshold to aid with defining genera in the family *Rhizobiaceae* (Fig. 2). The drop in the frequency distribution at ~86 % was also visible in scatterplots showing the relationships between cpAAI and either wpAAI or ANIb (Fig. S5). Notably, this corresponds nicely with a recent study that used a cpAAI threshold of 86 % in genus demarcation in the roseobacter group of the class *α-Proteobacteria* [26]. Using a cpAAI threshold of ~86 %, combined with the phylogeny of Fig. 1, we were able to largely preserve the current taxonomy of the family *Rhizobiaceae *, recovering the genera *Agrobacterium *, *Ciceribacter *, *Endobacterium *, *Ensifer* (as previously defined), *Gellertiella *, *Georhizobium *, *Mycoplana *, ‘* Neopararhizobium *’, *Peteryoungia *, *Pseudorhizobium *, *Pararhizobium* and *Shinella* (Figs 3 and S6). Such a threshold would, however, split the genera *Allorhizobium *, *Hoeflea *, *Martelella *, *Neorhizobium* and *Rhizobium sensu stricto* into two or more genera.

Both wpAAI and ANIb were correlated with cpAAI (Fig. S5), although neither relationship was linear. However, there was less support for the presence of genus-level drops in the wpAAI or ANIb frequency distributions (Figs S3 and S4). Nonetheless, the wpAAI frequency distribution density increased sharply below 76.5 %, while there was a sharp increase in the ANIb frequency distribution below 78.5 %. Although noisier, a wpAAI threshold of 76.5 % or a ANIb threshold of 78.5 % returned similar genus demarcations as did a cpAAI threshold of ~86 %, with a few exceptions (Figs S7–S10). In the case of wpAAI, the genus *Martelella* was recovered as a single genus, *Hoeflea* was split into fewer genera, and the separation between the genus *Ciceribacter* and its sister taxon was less clear. When using ANIb, *Shinella* and *Mycoplana* were combined as a single genus, *Allorhizobium* was split into three genera instead of two, the genus *Martelella* was recovered as a single genus, and the separation between the genus *Ciceribacter* and its sister taxon was less clear.

### Proposal for a framework for genus delineation in the family *Rhizobiaceae*

Based on the results summarized above, we propose that genera within the family *Rhizobiaceae* be defined as monophyletic groups (as determined by a phylogenetic reconstruction using a core-genome analysis approach; Fig. 1) separated from related species using a pairwise cpAAI threshold of approximately 86 % calculated as described in the methods. We specify ‘approximately 86%’ to provide some flexibility in the threshold to allow for differences in the evolution of each genus; for example, a cpAAI value of 85.7 % appears better suited for the genus *Pararhizobium *. We strongly recommend the use of cpAAI over wpAAI or ANIb due to (1) its natural agreement – by construction – with the core-genome gene phylogeny, (2) clearer gaps in its distribution of values among *Rhizobiaceae *, and (3) the fact that it would not be sensitive to the wide genome size variation within the *Rhizobiaceae *, notably due to the variation in presence of large mobile genetic elements, including symbiotic and tumor-inducing megaplasmids. We do not, however, propose that cpAAI serve as the sole information source for genus demarcation as nearly all biological rules have exceptions. We therefore propose that genus demarcation using a cpAAI threshold higher than 86 % can be justified by the presence of alternate genomic or phenotypic evidence (as proposed below for splitting of the genus *Ensifer *), while a lower cpAAI threshold may be appropriate when considering historical classifications of genera within the family.

### Taxonomic implications of the proposed framework

Following the criteria for genus demarcation outline above would notably lead to the formation of several new genera for species currently assigned to the genus *Rhizobium *, which is notoriously paraphyletic. They also imply that a few genera (* Neorhizobium *, *Allorhizobium *, *Martelella *, *Hoeflea *, and *Rhizobium sensu stricto*) may be candidates for division. We also note that there is a clear break in the distribution of cpAAI values at ~73 % to ~74 % that may represent an appropriate threshold for delimiting the family *Rhizobiaceae *. If adopted, this threshold would result in the genera *Martelella* and *Hoeflea* being transferred to their own families, while the genera *Georhizobium* and ‘* Neopararhizobium *’ would form another family. However, a proposal for family-level demarcations in the order *Rhizobiales* is outside the scope of this work.

### Proposal of a new genus encompassing the species *R. oryzae* and ‘* R. rhizosphaerae *’

In a recent study presenting a phylogeny of 571 *Rhizobiaceae* and *Aurantimonadaceae* strains (ML tree based on 155 concatenated core proteins) [28], the type strains of the species *R. oryzae* (* Allorhizobium oryzae *) [29] and ‘* R. rhizosphaerae *’ formed a well-delineated clade (with 100 % bootstrap support) that was clearly separated from the closest validly published genus type, i.e. *Pararhizobium giardinii* strain H152^T^. This pattern was also evident from an ML phylogeny of 797 *Rhizobiaceae* produced in another study based on the concatenation of 120 near-universal bacterial core genes [6]. The analyses presented in the current study further support the separation of the *R. oryzae */‘* R. rhizosphaerae *’ clade (*RoRr* clade; two species type strains) not only from the *Pararhizobium* clade (four species type strains), but also from a sister clade consisting solely of rhizobial strains from unnamed species (*unRhsp* clade; including *Rhizobium* sp. strains Leaf383, Leaf371, 9140 and NFR03) (Fig. 1); all three clades in the phylogenetic tree are supported by 100 % bootstrap values. All within-clade pairwise cpAAI values were above 85.7, 91 and 94 % for the *Pararhizobium* clade, *RoRr* clade, and the unnamed *Rhizobium* clade, respectively (Fig. 3). In contrast, all pairwise cpAAI values between the *RoRr* clade and the *Pararhizobium* or *unRhsp* clades were less than 81%, while pairwise cpAAI values between the *Pararhizobium* and *unRhsp* clades were below 83.5 % (Fig. 3). All three clades thus represent separate genera according to the criteria proposed above, and this remains true when the analysis is repeated with an expanded set of strains (Fig. S11). We therefore propose to define a new genus encompassing the *RoRr* clade, for which we propose the name *Xaviernesmea* (see below for formal description). As no strains belonging to the *unRhsp* clade have been deposited in any international culture collection, we leave the task of describing new species and genera within this clade to others who have access to these strains.

### Taxonomy of the ‘* R. aggregatum* complex’

The ‘* Rhizobium aggregatum* complex’ was initially identified as a sister taxon of the genus *Agrobacterium* [29], with subsequent work demonstrating that it is instead located on a clade neighbouring the genus *Allorhizobium* [13]. Moreover, the latter study suggested that ‘* R. aggregatum* complex’ includes members of the genus *Ciceribacter* and that it may represent a novel genus on the basis of phylogenetic and multiple OGRI data, although the authors advised that further investigation was required [13]. It was recently suggested that the ‘* R. aggregatum* complex’ be split into two genera [30]. It was proposed that *R. daejeonense *, *R. naphthalenivorans* and *R. selenitireducens* be transferred to the genus *Ciceribacter *, while *R. ipomoeae *, *Rhizobium rhizophilum *, *R. rosettiformans* and *R. wuzhouense* be transferred to the novel genus *Peteryoungia* along with the novel species ‘* Peteryoungia desertarenae *’ [30]; some of these changes have recently been validated [31].

The analyses presented in the current study included 13 strains belonging to the ‘* R. aggregatum* complex’ (Fig. 1). The genus demarcation framework proposed here supports the previous studies indicating that the ‘* R. aggregatum* complex’ is separate from the genus *Allorhizobium *. A group of seven species that included all *Peteryoungia* species present in our analysis (* Rhizobium aggregatum* DSM 1111^T^, ‘* Agrobacterium albertimagni *’ AOL15^T^, ‘*Rhizobium glycinendophyticum*’ CL12^T^, *Peteryoungia ipomoeae* shin9-1^T^, ‘* Peteryoungia rhizophila *’ 7209-2^T^, ‘* Peteryoungia rosettiformans *’ W3^T^ and ‘* Peteryoungia wuzhouensis *’ W44^T^) formed a monophyletic group with 100 % bootstrap support (Fig. 1). All pairwise cpAAI values within this group were >88 %, while all pairwise cpAAI values against the other six ‘* R. aggregatum* complex’ species were <84.7 % (Fig. 3). These results support the formation of the genus *Peteryoungia* [30], which should also include *R. aggregatum *, as well as ‘* A. albertimagni *’, and ‘*R. glycinendophyticum*’. We therefore propose that *R. aggregatum* be transferred to the genus *Peteryoungia* (see below for formal description). The species ‘* A. albertimagni *’ and ‘*R. glycinendophyticum*’ should also be transferred to *Peteryoungia* once their names are validly published.

The remaining six ‘* R. aggregatum* complex’ species formed a monophyletic group that could be further sub-divided into two clades. One clade corresponded to a group of three *Ciceribacter* species including the genus type strain, while the other clade contained *R. daejeonense* DSM 17795^T^, *C. naphthalenivorans* TSY03b^T^ and *C. selenitireducens* ATCC BAA-1503^T^ (Fig. 1). All within-group cpAAI values were >86.5 % while all between-group cpAAI values were ≤85.4 %, providing support for these two clades representing separate genera. However, the bootstrap support for the split of these two clades in the phylogeny is only 80 %, and the topology of the tree in this region (Fig. 1) differs from the tree reported by Rahi *et al*., wherein *R. daejeonense *, *C. naphthalenivorans* and *C. selenitireducens* were not monophyletic (see Fig. 2 of [30]). Overall, the data presented here are not in agreement with *R. daejeonense *, *C. naphthalenivorans* and *C. selenitireducens* belonging to the genus *Ciceribacter *. Instead, we propose this clade be referred to as the ‘* R. daejeonense* complex’ pending further study – enabled by the availability of additional genomes of strains belonging to these clades – to resolve whether these species belong to the genus *Ciceribacter* or whether they should be transferred to a novel genus.

### Proposal for the emendation of the genus *Sinorhizobium* as a distinct genus from *Ensifer*

Taxonomy of the genus *Ensifer */* Sinorhizobium* has been the subject of discussion since the early 2000s. The genus *Ensifer* was proposed in 1982 to describe *Ensifer adhaerens *, a bacterial predator [9]. Subsequently, the genus *Sinorhizobium* was proposed in 1988 when *Rhizobium fredii* was reclassified as *Sinorhizobium fredii* [10], which was followed by the emendation of this genus by de Lajudie *et al*. in 1994 [32]. In 2002, as the 16S rRNA gene sequence of *E. adhaerens* became available, the Subcommittee on the Taxonomy of *Agrobacterium* and *Rhizobium* (hereafter ‘the subcommittee’) of the International Committee on Systematics of Prokaryotes (ICSP) noted that this taxon is a part of *Sinorhizobium* [33]. Although the subcommittee pointed out that the name *Ensifer* has priority, conservation of the name *Sinorhizobium* was endorsed in contravention of the rules of the International Code of Nomenclature of Prokaryotes (ICNP). Neighbour-joining trees reconstructed from 16S rRNA gene sequences or partial *recA* gene sequences, together with phenotypic data, provided further data interpreted as supporting the synonymy and unification of the genera *Sinorhizobium* and *Ensifer *, leading Willems *et al*. to propose the new combination ‘* Sinorhizobium adhaerens *’ [34]. Accordingly, in their Request for an Opinion to the Judicial Commission, Willems *et al*. officially proposed to conserve the name *Sinorhizobium* [34]. As the primary argument for conservation of the name *Sinorhizobium *, the authors indicated that the name *Ensifer* would cause misunderstanding and confusion in the scientific community. A few months later, in a Request for an Opinion to the Judicial Commission, J. M. Young argued that *Ensifer *, not *Sinorhizobium *, was the valid name for the unified genus, as *Ensifer* had priority [35]. At the same time, J. M. Young emended the description of the genus *Ensifer *, and transferred previously described *Sinorhizobium* species to this genus [35]. The Judicial Commission of the ICSP (Judicial Opinion 84) later confirmed that *Ensifer* had priority over *Sinorhizobium *, pointed out that the name ‘* Sinorhizobium adhaerens *’ is not validly published, and supported the transfer of members of the genus *Sinorhizobium* to *Ensifer* [36]. In this Opinion, it was claimed that the transfer of the members of the genus *Sinorhizobium* to the genus *Ensifer* would not cause confusion. The subcommittee, however, disagreed with this justification [37]. J. M. Young criticized these actions of the subcommittee [38], which was also later acknowledged by Tindall [39]. As predicted by Willems *et al*. [34], adoption of the genus name *Ensifer* continues to be met with resistance from many rhizobiologists [40].

Earlier phylogenetic studies noted that *E. adhaerens* was an outgroup of the genus *Ensifer* [41], providing some support that *E. adhaerens* represented a distinct genus; however, it was suggested that further evidence would be required prior to redefining genera within this clade [41]. Significant phylogenomic and phenotypic data now exists providing strong evidence that the genera *Ensifer* and *Sinorhizobium* as defined Casida 1982 [9] and Chen *et al*. 1988 [10], respectively, refer to closely related, yet separate, taxa. At least seven studies, including the Genome Taxonomy Database, have presented phylogenetic trees containing two well-defined clades within the genus *Ensifer* [21, 28, 42–46]. These phylogenies were built on the basis of gene (up to 1652 genes) or protein (up to 155 proteins) sequences using ML or Bayesian inference analysis approaches, indicating that the observed clades are robust to the choice of phylogenetic approach. Notably, our recent study presents an ML phylogeny where the genus *Ensifer* is split into two clades of 12 and 20 genospecies with 100 % bootstrap support for the split, which we then defined as the ‘nonsymbiotic’ and ‘symbiotic’ clades, respectively [21]. The split is also observed in an ML phylogeny of the 16S rRNA genes of the same strains, with 62 % bootstrap support (Fig. S12). We similarly see a split of the genus *Ensifer* into two clades of three species type strains (including *E. adhaerens* Casida A^T^, the type strain of the type species of the genus *Ensifer* Casida 1982) and 12 species type strains (including *E. fredii* USDA 205^T^, the type strain of the type species of the genus *Sinorhizobium* Chen *et al*. 1988) in our core-genome gene phylogeny, with 100 % bootstrap support (Fig. 1), representing the nonsymbiotic and symbiotic clades, respectively. However, all between-clade cpAAI values were above the suggested 86 % threshold as a baseline criterion for genus delimitation. Despite this, and following our proposed framework, we argue that there is sufficient other genomic and phenotypic data supporting the division of this genus (cf. Figs 3–5 of [21]). We describe the distinctive traits and respective synapomorphies of these clades in Table 1 and below.

**Table 1.** Characteristics differentiating the previously-defined nonsymbiotic and symbiotic clades of the genus *Ensifer *, corresponding to the emended genera *Ensifer* and *Sinorhizobium *, respectively

Characteristic	Nonsymbiotic clade (emended genus *Ensifer *)	Symbiotic clade (emended genus *Sinorhizobium *)	Reference
GANTC sites per kb	0.9–1.3 (mean: 1.06)	1.5 to 1.8 (mean: 1.70)	[47]
Number of CDS	5816–7682 (mean: 6876)	5516 to 8629 (mean: 6550)	[21]
Ribosomal RNA operons	5	3	[21]
Carries *nod* and *nif* genes	No*	Yes	[21]
Bacterial predation ability	Yes	No	[9, 52, 53]
Division by budding	Yes	No	[50]
Growth in unmodified LB medium	Yes	Poor	[21]
Starch hydrolysis	Yes	No	[51]
Growth at 37 ˚C	No (generally)	Yes (generally)	[21]
Fatty acids	More C_16 : 0_ 3OH	More C_18 : 1_ *ω*9*c*	[51]
Carbon sources used (Biolog PM1/PM2)	69–87 (mean: 81)	50–81 (mean: 65)	[21]
pH tolerance (Biolog PM9)	Better low pH tolerance	Better high pH tolerance	[21]

*Except for the species *E. sesbaniae*, whose nine strains are legume symbionts.

The genome-wide frequency of the pentanucleotide GANTC is higher in all genomes of the symbiotic clade compared to all genomes of the nonsymbiotic clade, with a statistically significant average difference of 60 % (1.70 vs 1.06 GANTC sites per kb, *P­*-value<1×10^−10^ using a two-sample *t*-test) [47]. As the GANTC motif is methylated by the highly conserved cell cycle-regulated methyltransferase CcrM [48, 49], this difference may reflect an important difference in the cell cycle biology of these two clades [47]. Indeed, species of the nonsymbiotic clade (* E. adhaerens* and *E. morelensis *) are capable of division by budding, unlike species of the symbiotic clade [50]. It has also been shown that the ability to hydrolyze starch [51] and robustly grow in LB broth lacking Ca^2+^ and Mg^2+^ ion supplementation [21] is specific to the nonsymbiotic clade. Stress tolerance of the two clades also differs (based on an analysis of 10 representative strains), with strains of the nonsymbiotic clade generally being more tolerant to alkaline conditions while strains of the symbiotic clade were generally more acid-tolerant and heat-tolerant [21]. Although many catabolic abilities could be found in at least a subset of each clade, which is unsurprising given both clades have open pangenomes, species of the nonsymbiotic clade are capable of catabolizing an average of 81 (out of 190 tested) carbon sources compared to an average of 65 for the symbiotic clade [21]. These differences in general phenotypic traits, together with the additional genomic and phenotypic differences outlined in Table 1, indicate marked differences in the biology of strains from these two clades. Indeed, at least two genospecies of the nonsymbiotic clade have been described as bacterial predators [9, 52, 53], a lifestyle that has not been attributed to any members of the symbiotic clade. Moreover, these two clades display significant differences in relation to their interactions with plant species, specifically, a biased distribution of the *nod* and *nif* genes required for establishment of nitrogen-fixing symbiosis with legumes [21, 45]. A recent study showed that whereas the core *nodABC* and *nifHDK* genes were present in strains from all 20 genospecies of the symbiotic clade, they were observed in just one of the 12 genospecies belonging the nonsymbiotic clade (* E. sesbaniae *, with all nine reported strains, isolated from three different geographic origins, being symbiotic) [21, 51]. Symbiotic traits are linked to the presence of an accessory megaplasmid in the genome, and thus should not be considered relevant in delineating taxa [7]. However, this almost unique ability of genomes from the symbiotic clade to host symbiotic megaplasmids with respect to their relatives from the nonsymbiotic clade likely reflects differences in their genetic background. These discrepancies in symbiotic potential could thus be interpreted as a further marker of differentiated biology between these two clades.

Taken together, these genomic and phenotypic data suggest that the organisms in these two clades significantly differ in their biology and ecology, reminiscent of the stable ecotype model for bacterial species [54]. Notably, the type species of the genus *Ensifer* (* E. adhaerens *) is found within the nonsymbiotic clade, while the original type species of the genus *Sinorhizobium* (* S. fredii *) is found within the symbiotic clade. Given we established the taxonomic position of these type species-containing clades to be well separated, we argue that the proposal of Willems *et al*. [34] to unify the genera *Ensifer* Casida 1982 and *Sinorhizobium* Chen *et al*. 1988, and the Judicial Opinion 84 enacting the transfer of the members of the genus *Sinorhizobium* to the genus *Ensifer* [36], are no longer supported. Instead, we propose that *Ensifer* Casida 1982 and *Sinorhizobium* Chen *et al*. 1988 refer to closely related sister genera, of which *Ensifer* and *Sinorhizobium *, respectively, are the legitimate names in accordance with Rules 51a and 23a of the ICNP [55]. We note that the subcommittee has previously indicated support for this proposal [56], while also stating they are not in favour of creating subgenera for these taxa [40]. Formal genus and species emendations and circumscriptions are provided below.

### Taxonomy of the genus *Neorhizobium*

More study is required to resolve the taxonomic relationships between the ‘*Neorhizobium sensu stricto*’ clade (Fig. 1) – which includes *N. vignae *, *N. alkalisolii*, *N. hautlense*, *N. galegae* and *N. tomejilense *, as well as *Rhizobium petrolearium* – and related taxa. The core-genome gene phylogeny (Fig. 1) and the cpAAI data (Fig. 3) suggest that ‘*Neorhizobium lilium*’ represents a new genus, as does the clade formed by *Neorhizobium* sp. NCHU2750, *Rhizobium smilacinae *, and *Rhizobium cellulosilyticum *. However, because bootstrap values provided only moderate support for the topology of the tree in the extended *Neorhizobium* clade and the clades were not well-resolved by the cpAAI data, we defer the proposal of new genera until publication of further genomic evidence.

### Additional taxonomic implications of the proposed framework for genus delimitation

*R. petrolearium* DSM 26482^T^ formed a clade with ‘*Neorhizobium sensu stricto*’ (Fig. 1). Pairwise cpAAI values were all >90 % when *R. petrolearium* was compared against ‘*Neorhizobium sensu stricto*’ species type strains (Fig. 3). We therefore propose that *R. petrolearium* be transferred to the genus *Neorhizobium* (see below for formal description).

*Rhizobium tarimense* CCTC AB 2011022^T^ formed a clade with the genus *Pseudorhizobium* (Fig. 1). Pairwise cpAAI values were all >88 % when *R. tarimense* was compared against the four *Pseudorhizobium* species type strains, but <85 % when compared against all other species (Fig. 3). We therefore propose that *R. tarimense* be transferred to the genus *Pseudorhizobium* (see below for formal description).

*Rhizobium arenae* MIM27^T^ formed a clade with the genus *Pararhizobium *. Pairwise cpAAI values were 87.4, 87.4 and 85.7 % when *R. arenae* was compared against the three *Pararhizobium* species type strains, but <84 % when compared against all other species (Fig. 3). We therefore propose that *R. arenae* be transferred to the genus *Pararhizobium* (see below for formal description).

*Rhizobium azooxidifex* DSM 100211^T^ formed a clade with ‘* Mycoplana subbaraonis *’ JC85^T^ and *Mycoplana dimorpha* DSM 7138^T^ (Fig. 1). Pairwise cpAAI values between these three species type strains were all >89 %, while cpAAI values against strains outside of this clade were all <85 % (Fig. 3). We therefore propose that *R. azooxidifex* be transferred to the genus *Mycoplana* (see below for formal description).

*Rhizobium yantingense* CCTCC AB 2014007^T^ formed a clade with *Endobacterium cereale* (Fig. 1). The pairwise cpAAI value between these two species was >95 %, while cpAAI values against strains outside of this clade were all <84 % (Fig. 3). We therefore propose that *R. yantingense* be transferred to the genus *Endobacterium* (see below for formal description).

To confirm the distinct taxonomic positions of the above-mentioned species and support their transfer to the respective genera, we compared them to other genus members using ANIb and dDDH indices. These indices are regarded as standard measures of relatedness between prokaryotic species that were widely used for species delimitation [57, 58]. In all cases, the obtained values were clearly below the thresholds for species delimitation (95–96 % for ANI or 70 % for DDH) (Datasets S2 and S3), confirming the authenticity of these species.

In addition, the following species are candidates as type species for new genera: ‘*R. album*’, *R. populii*, *R. borbori* and *R. halophytocola *. The reclassification of *R. album* into a new genus was also suggested by Young *et al*. [6]. Moreover, *R. helianthi* CGMC 1.12192^T^, *R. rhizoryzae* DSM 29514^T^ and *Allorhizobium pseudoryzae* DSM 19479^T^ formed a monophyletic group as a sister taxon to the genus *Endobacterium* (Fig. 1). This clade of three species type strains is another candidate for reclassification as a new genus as pairwise cpAAI values within the clade were all >86.5 % while all cpAAI values with species outside of the clade were all <83 % (Fig. 3).

## Description of *Xaviernesmea* gen. nov.

*Xaviernesmea* (gza.vje.nem’e.a.; N.L. fem. n., in honour of Dr. Xavier Nesme, taxonomist of agrobacteria and rhizobia who pioneered the use of reverse ecology approaches to infer the ecology of *Agrobacterium* genomic species from comparative genomic analyses [8]).

Cells are Gram-negative, rod-shaped, and aerobic. Oxidase- and catalase-positive. Can utilize adonitol, raffinose and succinic acid. The pH range for growth is pH 5.0–11.0 [59]. The G+C content of the genomic DNA is in the range 62.8–64.7 mol%. The genus *Xaviernesmea* has been separated from other *Rhizobiaceae* genera based on a core-genome phylogeny and whole- and core-proteome relatedness indices (wpAAI and cpAAI).

The type species is *Xaviernesmea oryzae*.

## Description of *Xaviernesmea oryzae* comb. nov.

*Xaviernesmea oryzae* (o.ry’zae. L. gen. fem. n. *oryzae,* of rice, referring to the host of isolation of the type strain).

Basonym: *Rhizobium oryzae* Peng *et al*. 2008 [60].

Homotypic synonym: *Allorhizobium oryzae* (Peng et al., 2008) Mousavi *et al*. 2015.

The description is as provided by Peng *et al*. 2008 [60] and Mousavi *et al*. 2015 [29]. *X. oryzae* can be differentiated from other species of the genus *Xaviernesmea* based on OGRI calculations (ANI and dDDH). The genomic G+C content of the type strain is 62.8 mol%. Its approximate genome size is 5.39 Mbp.

The type strain is Alt 505^T^ (=LMG 24253^T^=CGMCC 1.7048^T^), isolated from *Oryza alta* growing in the Wild Rice Core Collection Nursery of South China Agricultural University. The NCBI RefSeq Assembly accession number for the genome sequence is GCF_900109605.1.

## Description of *Xaviernesmea rhizosphaerae* sp. nov.

*Xaviernesmea rhizosphaerae* (rhi.zo.sphae’rae. N.L. gen. fem. n. *rhizosphaerae*, of the rhizosphere, referring to host plant compartment of isolation of the type strain).

The description is as provided by Zhao *et al*. [59]. *X. rhizosphaerae* can be differentiated from other species of the genus *Xaviernesmea* based on OGRI calculations (ANI and dDDH). The genomic G+C content of the type strain is 64.7 mol%. Its approximate genome size is 5.18 Mbp.

The type strain is MH17^T^ (=ACCC 19963^T^=KCTC 52414^T^), which was isolated from the roots of rice collected from Beijing, PR China. The NCBI RefSeq assembly accession number for the genome sequence is GCF_001938945.1. We note that the name ‘* Rhizobium rhizosphaerae *’ that was proposed in the original publication [59] has yet to be validly published.

## Emended description of the genus *Ensifer* Casida 1982

The description is as given by Casida 1982 [9] with the following emendations. The optimal growth temperature is 27–28 °C. Some species can grow at 37 °C. Capable of growth in unmodified lysogeny broth (LB). Capable of hydrolysing starch. Resistant to multiple antibiotics including ampicillin and erythromycin. The genomic G+C content is ~61–63 mol%. The genomic GANTC pentanucleotide frequency is ~0.9–1.3 sites per kb. Most strains carry five rRNA operons. The genus can be differentiated from other genera based on core-genome gene phylogenies.

The type species is *Ensifer adhaerens *.

The emended genus contains the species *E. adhaerens *, *E. morelensis* and *E. sesbaniae *.

The species *E. alkalisoli *, *E. americanum *, *E. arboris *, *E. fredii *, *E. garamanticum*, *E. glycinis *, *E. kostiensis *, *E. kummerowiae *, *E. medicae *, *E. meliloti *, *E. mexicanum*, *E. numidicus *, *E. psoraleae *, *E. saheli *, *E. sofinae*, *E. sojae* and *E. terangae* are transferred to the genus *Sinorhizobium *.

## Emended description of the genus *Sinorhizobium* Chen et al. 1988 emend. de Lajudie *et al*. 1994

The description is as given by de Lajudie *et al*. [32] with the following emendations, drawing also from Young [35]. The optimal growth temperature is 25–33 °C, but some strains can grow at 12 °C and others can grow at 44 °C. Optimum pH is 6–7, but some strains can grow at pH 5.0 and others at pH 10.0. Starch is not utilized. Ammonium salts, nitrate, nitrite, and many amino acids can serve as nitrogen sources for most strains. Most strains produce cytochrome oxidase and catalase. The genomic G+C content is ~61–64 mol%. The genomic GANTC pentanucleotide frequency is ~1.5–1.8 sites per kb. Most strains carry three rRNA operons. The genus can be differentiated from other genera based on core-genome gene phylogenies.

The type species is *Sinorhizobium fredii *.

The emended genus contains the species *S. alkalisoli*, *S. americanum *, *S. arboris *, *S. fredii *, *S. garamanticum*, *S. glycinis*, *S. kostiense *, *S. kummerowiae *, *S. medicae *, *S. meliloti *, *S. mexicanum*, *S. numidicum*, *S. psoraleae*, *S. saheli *, *S. sofinae*, *S. sojae* and *S. terangae *.

## Description of *Sinorhizobium alkalisoli* comb. nov.

*Sinorhizobium alkalisoli* (al.ka.li.so’li. N.L. neut. n. *alkali*, alkali (from Arabic al-qaliy); L. neut. n. *solum*, soil; N.L. gen. neut. n. *alkalisoli*, of alkaline soil, referring to the saline-alkali soil where the bacterium was isolated).

Basonym: *Ensifer alkalisoli* Li *et al*. 2016.

The description is as provided by Li *et al*. 2016 [61]. *S. alkalisoli* can be differentiated from other species of the genus *Sinorhizobium* based on OGRI calculations (ANI and dDDH). The genomic G+C content of the type strain is 62.2 mol%. Its approximate genome size is 6.13 Mbp.

The type strain is YIC4027^T^ (=HAMBI 3655^T^=LMG 29286^T^). The NCBI RefSeq assembly accession number for the genome sequence is GCF_001723275.1.

## Emended description of *Sinorhizobium americanum* Toledo *et al*. 2004

*Sinorhizobium americanum* (a.me.ri.ca’num. N.L. neut. adj. *americanum*, American, referring to the isolation of the type strain from the Colorado Plateau).

Homotypic synonym: *Ensifer americanus* (Toledo *et al*. 2004) Wang *et al*. 2015 emend. Hördt *et al*. 2020.

The description is as provided by Hördt et al. 2020 [5]. *S. americanum* can be differentiated from other species of the genus *Sinorhizobium* based on OGRI calculations (ANI and dDDH). The genomic G+C content of the type strain is 62.3 mol%. Its approximate genome size is 6.75 Mbp.

The type strain is CFNEI 156^T^ (=ATCC BAA-532^T^=CIP 108390^T^=DSM 15007^T^). The NCBI RefSeq assembly accession number for the genome sequence is GCF_001651855.1.

## Emended description of *Sinorhizobium arboris* Nick *et al*. 1999

*Sinorhizobium arboris* (ar’bo.ris. L. fem. n. arbour, tree; L. gen. fem. n. *arboris*, of a tree).

Homotypic synonym: *Ensifer arboris* (Nick *et al*. 1999) Young 2003 emend. Hördt *et al*. 2020.

The description is as provided by Hördt *et al*. 2020 [5]. *S. arboris* can be differentiated from other species of the genus *Sinorhizobium* based on OGRI calculations (ANI and dDDH). The genomic G+C content of the type strain is 62.0 mol%. Its approximate genome size is 6.85 Mbp.

The type strain is HAMBI 1552^T^ (=ATCC BAA-226^T^=DSM 13375^T^=LMG 14919^T^=NBRC 100383^T^=TTR 38^T^). The NCBI RefSeq assembly accession number for the genome sequence is GCF_000427465.1.

## Emended description of *Sinorhizobium fredii* (Scholla and Elkan 1984) Chen *et al*. 1988

*Sinorhizobium fredii* (fred'i.i. N.L. gen. neut. n. *fredii*, of Fred, named after of E.B. Fred).

Homotypic synonym: *Ensifer fredii* (Scholla and Elkan 1984) Young 2003 emend. Hördt *et al*. 2020.

Heterotypic synonym: *Ensifer xinjiangensis* (Chen *et al*. 1988) Young 2003 [43]

The description is as provided by Hördt *et al*. 2020 [5]. *S. fredii* can be differentiated from other species of the genus *Sinorhizobium* based on OGRI calculations (ANI and dDDH). The genomic G+C content of the type strain is 62.2 mol%. Its approximate genome size is 7.15 Mbp.

The type strain is USDA 205^T^ (=ATCC 35423^T^=CCUG 27877^T^=DSM 5851^T^=HAMBI 2075^T^=ICMP 11139^T^=IFO 14780^T^=JCM 20967^T^=LMG 6217^T^=NBRC 14780^T^=NRRL B-14241^T^=NRRL B-14594^T^=PRC 205^T^). The NCBI RefSeq Assembly accession number for the genome sequence is GCF_001461695.1.

## Description of *Sinorhizobium garamanticum* comb. nov.

*Sinorhizobium garamanticum* (ga.ra.man’ti.cum. N.L. neut. adj. *garamanticum*, pertaining to Garamante, Garamantian, the country of Garamantes, from which the strains were isolated).

Basonym: *Ensifer garamanticus* Merabet *et al*. 2010.

The description is as provided by Merabet *et al*. 2010 [62]. *S. garamanticum* can be differentiated from other species of the genus *Sinorhizobium* by phylogenetic analysis based on several housekeeping (*recA*, *glnA*, *gltA*, *thrC* and *atpD*) genes and 16S rRNA gene sequencing. The genomic G+C content of the type strain is approximately 62.4 mol% (HPLC).

The type strain is ORS 1400^T^ (=CIP 109916^T^=LMG 24692^T^).

## Description of *Sinorhizobium glycinis* comb. nov.

*Sinorhizobium glycinis* (gly.ci’nis. N.L. gen. n. *glycinis*, of the botanical genus *Glycine*, the soybean, named for its nodulation characteristics and symbiotic genes).

Basonym: *Ensifer glycinis* Yan *et al*. 2016.

The description is as provided by Yan *et al*. 2016 [63]. *S. glycinis* can be differentiated from other species of the genus *Sinorhizobium* based on OGRI calculations (ANI and dDDH). The genomic G+C content of the type strain is 62.4 mol%. Its approximate genome size is 6.04 Mbp.

The type strain is CCBAU 23380^T^ (=HAMBI 3645^T^=LMG 29231^T^). The NCBI RefSeq assembly accession number for the genome sequence is GCF_001651865.1.

## Emended description of *Sinorhizobium kostiense* Nick *et al*. 1999

*Sinorhizobium kostiense* (kos.ti.en’se. N.L. neut. adj. *kostiense*, pertaining to Kosti, the region in Sudan where most of these organisms have been isolated).

Homotypic synonym: *Ensifer kostiensis* (Nick *et al*. 1999) Young 2003.

The description is as provided by Nick *et al*. 1999 [64]. *S. kostiense* can be differentiated from other species of the genus *Sinorhizobium* based on OGRI calculations (ANI and dDDH). The genomic G+C content of the type strain is 61.7 mol%. Its approximate genome size is 6.33 Mbp.

The type strain is DSM 13372^T^ (=ATCC BAA-227^T^=HAMBI 1489^T^=LMG 15613^T^=LMG 19227^T^=NBRC 100382^T^=TTR 15^T^). The NCBI RefSeq assembly accession number for the genome sequence is GCF_017874595.1.

## Emended description of *Sinorhizobium kummerowiae* Wei *et al*. 2002

*Sinorhizobium kummerowiae* (kum.me.ro’wi.ae. N.L. gen. fem. n. *kummerowiae*, of *Kummerowia*, a genus of leguminous plants, referring to the host from which the bacterium was isolated).

Homotypic synonym: *Ensifer kummerowiae* (Wei *et al*. 2002) Young 2003.

The description is as provided by Wei *et al*. 2002 [65]. *S. kummerowiae* can be differentiated from other species of the genus *Sinorhizobium* by phylogenetic analysis based on 16S rRNA gene sequences. The genomic G+C content of the type strain is approximately 61.6 mol% (*Tm*).

The type strain is CCBAU 71714^T^ (=CGMCC 1.3046^T^=CIP 108026^T^=NBRC 100799^T^).

## Emended description of *Sinorhizobium medicae* Rome *et al*. 1996

*Sinorhizobium medicae* (me’di.cae. L. gen. fem. n. *medicae*, of/from lucerne (plant belonging to the genus *Medicago*).

Homotypic synonym: *Ensifer medicae* (Rome *et al.* 1996) Young 2003.

The description is as provided by Rome *et al*. 1996 [66]. *S. medicae* can be differentiated from other species of the genus *Sinorhizobium* based on OGRI calculations (ANI and dDDH). The genomic G+C content of the type strain is 61.2 mol%. Its approximate genome size is 6.53 Mbp.

The type strain is A 321^T^ (=11-3 21 a^T^=HAMBI 2306^T^=ICMP 13798^T^=LMG 19920^T^=NBRC 100384^T^=USDA 1037^T^). The NCBI RefSeq assembly accession number for the genome sequence is GCF_009599935.1.

## Emended description of *Sinorhizobium meliloti* (Dangeard 1926) de Lajudie *et al*. 1994

*Sinorhizobium meliloti* (me.li.lo’ti. N.L. masc. n. *Melilotus*, generic name of sweet clover; N.L. gen. masc. n. *meliloti*, of *Melilotus*).

Homotypic synonym: *Ensifer meliloti* (Dangeard 1926) Young 2003.

The description is as provided by de Lajudie *et al*. 1994 [32]. *S. meliloti* can be differentiated from other species of the genus *Sinorhizobium* based on OGRI calculations (ANI and dDDH). The genomic G+C content of the type strain is 62.0 mol%. Its approximate genome size is 7.34 Mbp.

The type strain is USDA 1002^T^ (=ATCC 9930^T^=CCUG 27879^T^=CFBP 5561^T^=DSM 30135^T^=HAMBI 2148^T^=ICMP 12623^T^=IFO 14782^T^=JCM 20682^T^=LMG 6133^T^=NBRC 14782^T^=NCAIM B.01520^T^=NRRL L-45^T^=NZP 4027^T^). The NCBI RefSeq assembly accession number for the genome sequence is GCF_009601385.1.

## Description of *Sinorhizobium mexicanum* comb. nov.

*Sinorhizobium mexicanum* (me.xi.ca’num. N.L. neut. adj. *mexicanum*, of or belonging to Mexico, where the strains were isolated).

Basonym: *Ensifer mexicanus* Lloret *et al*. 2011.

The description is as provided by Lloret *et al*. 2011 [67]. *S. mexicanum* can be differentiated from other species of the genus *Sinorhizobium* based on OGRI calculations (ANI and dDDH). The genomic G+C content of the type strain is 61.4 mol%. Its approximate genome size is 7.14 Mbp.

The type strain is ITTG R7^T^ (=ATCC BAA-1312^T^=CFN ER1001^T^=CIP 109033^T^=DSM 18446^T^=HAMBI 2910^T^). The NCBI RefSeq assembly accession number for the genome sequence is GCF_013488225.1.

## Description of *Sinorhizobium numidicum* comb. nov.

*Sinorhizobium numidicum* (nu.mi’di.cum. N.L. neut. adj. *numidicum*, pertaining to the country of Numidia, Numidian, the Roman denomination of the region in North Africa from which the majority of the organisms were isolated).

Basonym: *Ensifer numidicus* Merabet *et al*. 2010.

The description is as provided by Merabet *et al*. 2010 [62]. *S. numidicus* can be differentiated from other species of the genus *Sinorhizobium* by phylogenetic analysis based on several housekeeping (*recA*, *glnA*, *gltA*, *thrC* and *atpD*) genes and 16S rRNA gene sequencing. The genomic G+C content of the type strain is approximately 62.8 mol% (HPLC).

The type strain is ORS 1407^T^ (=CIP 109850^T^=LMG 27395^T^).

## Description of *Sinorhizobium psoraleae* comb. nov.

*Sinorhizobium psoraleae* (pso.ra.le’ae N.L. gen. fem. n. *psoraleae*, of *Psoralea*, referring to the main host of the species).

Basonym: *Ensifer psoraleae* Wang *et al*. 2013.

The description is as provided by Wang *et al*. 2013 [51]. *S. psoraleae* can be differentiated from other species of the genus *Sinorhizobium* based on OGRI calculations (ANI and dDDH). The genomic G+C content of the type strain is 61.3 mol%. Its approximate genome size is 7.43 Mbp.

The type strain is CCBAU 65732^T^ (=HAMBI 3286^T^=LMG 26835^T^). The NCBI RefSeq assembly accession number for the genome sequence is GCF_013283645.1.

## Emended description of *Sinorhizobium saheli* de Lajudie *et al*. 1994

*Sinorhizobium saheli* (sa’hel.i. N.L. gen. neut. n. *saheli*, of the Sahel, the region in Africa from which they were isolated).

Homotypic synonym: *Ensifer saheli* (de Lajudie *et al*. 1994) Young 2003 emend. Hördt *et al*. 2020.

The description is as provided by Hördt *et al*. 2020 [5]. *S. saheli* can be differentiated from other species of the genus *Sinorhizobium* based on OGRI calculations (ANI and dDDH). The genomic G+C content of the type strain is 63.6 mol%. Its approximate genome size is 5.99 Mbp.

The type strain is LMG 7837^T^ (=ATCC 51690^T^=DSM 11273^T^=HAMBI 215^T^=ICMP 13648^T^=NBRC 100386^T^=ORS 609^T^). The NCBI RefSeq assembly accession number for the genome sequence is GCF_001651875.1.

## Description of *Sinorhizobium shofinae* comb. nov.

*Sinorhizobium shofinae* (sho.fi’nae. N.L. fem. gen. n. *shofinae* from Shofine, a company name, referring to the fact that the type strain of this species was isolated from root nodule of soybean grown in the farm of the company, Shandong Shofine Seed Technology Company Ltd., located in Jiaxiang County, Shandong Province of China).

Basonym: *Ensifer shofinae* Chen *et al*. 2017 emend. Hördt *et al*. 2020.

The description is as provided by Hördt *et al*. 2020 [5]. *S. shofinae* can be differentiated from other species of the genus *Sinorhizobium* based on OGRI calculations (ANI and dDDH). The genomic G+C content of the type strain is 59.9 mol%. Its approximate genome size is 6.21 Mbp.

The type strain is CCBAU 251167^T^ (=HAMBI 3507^T^=LMG 29645^T^=ACCC 19939^T^). The NCBI RefSeq assembly accession number for the genome sequence is GCF_001704765.1.

## Description of *Sinorhizobium sojae* comb. nov.

*Sinorhizobium sojae* (so'ja.e. N.L. gen. n. *sojae*, of soja, of soybean, referring to the source of the first isolates).

Basonym: *Ensifer sojae* Li *et al*. 2011 emend. Hördt *et al*. 2020.

The description is as provided by Hördt *et al*. 2020 [5]. *S. sojae* can be differentiated from other species of the genus *Sinorhizobium* based on OGRI calculations (ANI and dDDH). The genomic G+C content of the type strain is 60.9 mol%. Its approximate genome size is 6.09 Mbp.

The type strain is CCBAU 5684^T^ (=DSM 26426^T^=HAMBI 3098^T^=LMG 25493^T^). The NCBI RefSeq assembly accession number for the genome sequence is GCF_000261485.1.

## Emended description of *Sinorhizobium terangae* de Lajudie *et al.* 1994

*Sinorhizobium terangae* [te’ran.gae. N.L. n. *terengae*, hospitality (from West African Wolof n. terenga, hospitality); N.L. gen. n. *terangae*, of hospitality, intended to mean that this species is isolated from different host plants].

Homotypic synonym: *Ensifer terangae* (de Lajudie *et al*. 1994) Young 2003.

The description is as provided by de Lajudie *et al*. 1994 [32]. *S. terangae* can be differentiated from other species of the genus *Sinorhizobium* based on OGRI calculations (ANI and dDDH). The genomic G+C content of the type strain is 61.4 mol%. Its approximate genome size is 6.79 Mbp.

The type strain is SEMIA 6460^T^ (=ATCC 51692^T^=DSM 11282^T^=HAMBI 220^T^=ICMP 13649^T^=LMG 7834^T^=NBRC 100385^T^=ORS 1009^T^). The NCBI RefSeq assembly accession number for the genome sequence is GCF_014197705.1.

## Description of *Endobacterium yantingense* comb. nov.

*Endobacterium yantingense* (yan. ting. en’se. N.L. neut. adj. *yantingense* referring to Yanting district, Sichuan Province, PR China, where the organism was isolated).

Basonym: *Rhizobium yantingense* Chen *et al*. 2015.

The description is as provided by Chen *et al*. 2015 [68]. *E. yantingense* can be differentiated from another species of the genus *Endobacterium* (* Endobacterium cereale* corrig. Menéndez *et al*. 2021) based on OGRI calculations (ANI and dDDH). The genomic G+C content of the type strain is 59.5 mol%. Its approximate genome size is 5.82 Mbp.

The type strain is H66^T^ (=CCTCC AB 2014007^T^=LMG 28229^T^), which was isolated from the surfaces of weathered rock (purple siltstone) in Yanting (Sichuan, PR China). The JGI IMG accession number for the genome sequence is Ga0196656.

## Description of *Neorhizobium petrolearium* comb. nov.

*Neorhizobium petrolearium* (pe.tro.le.a’ri.um. L. fem. n. *petra*, rock; L. neut. *olearium* related to oil, of or belonging to oil; N.L. neut. adj. *petrolearium* related to mineral oil).

Basonym: *Rhizobium petrolearium* Zhang *et al*. 2012.

The description is as provided by Zhang *et al*. 2012 [69]. *N. petrolearium* can be differentiated from another species of the genus *Neorhizobium* based on OGRI calculations (ANI and dDDH). The genomic G+C content of the type strain is 60.5 mol%. Its approximate genome size is 6.97 Mbp.

The type strain, SL-1^T^ (=ACCC 11238^T^=KCTC 23288^T^), was isolated from petroleum-contaminated sludge samples in Shengli oilfield, Shandong Province, China. The JGI IMG accession number for the genome sequence is Ga0196653.

## Description of *Pararhizobium arenae* comb. nov.

*Pararhizobium arenae* (a.re’nae. L. fem. gen. n. *arenae* of sand, the isolation source of the type strain).

Basonym: *Rhizobium arenae* Zhang *et al*. 2017.

The description is as provided by Zhang *et al*. 2017 [70]. *P. arenae* can be differentiated from another species of the genus *Pararhizobium* based on OGRI calculations (ANI and dDDH). The genomic G+C content of the type strain is 59.8 mol%. Its approximate genome size is 4.94 Mbp.

The type strain is MIM27^T^ (=KCTC 52299^T^=MCCC 1K03215^T^), isolated from sand of the Mu Us Desert, PR China. The NCBI RefSeq Assembly accession number for the genome sequence is GCF_001931685.1.

## Description of *Peteryoungia aggregata* comb. nov.

*Peteryoungia aggregata* (ag.gre.ga’ta. L. fem. part. adj. *aggregata*, joined together, referring to the frequent formation of rosettes).

Basonym: *Rhizobium aggregatum* Kaur *et al*. 2011 [71].

Homotypic synonym: *Blastobacter aggregatus* Hirsch and Muller 1986 [72].

The description is as provided by Kaur *et al*. 2011 [71]. *P. aggregata* can be differentiated from another species of the genus *Peteryoungia* based on OGRI calculations (ANI and dDDH). The genomic G+C content of the type strain is 62.7 mol%. Its approximate genome size is 4.81 Mbp.

The type strain is IFAM 1003^T^ (= DSM 1111^T^=ATCC 43293^T^). The JGI IMG accession number for the genome sequence is Ga0196658.

## Description of *Pseudorhizobium tarimense* comb. nov.

*Pseudorhizobium tarimense* (ta.rim.en’se. N.L. neut. adj. *tarimense*, pertaining to Tarim basin in Xinjiang Uyghur autonomous region of China, where the type strain was isolated).

Basonym: *Rhizobium tarimense* Turdahon *et al*. 2013.

The description is as provided by Turdahon *et al*. 2013 [73]. *P. tarimense* can be differentiated from other species of the genus *Pseudorhizobium* based on OGRI calculations (ANI and dDDH). The genomic G+C content of the type strain is 61.2 mol%. Its approximate genome size is 4.83 Mbp.

The type strain is CCTCC AB 2011011^T^ (=NRRL B-59556^T^=PL-41^T^). The JGI IMG accession number for the genome sequence is Ga0196649.

## Description of *Mycoplana azooxidifex* comb. nov.

*Mycoplana azooxidifex* [a.zo.o.xi’di.fex. N.L. neut. n. *azooxidum*, dinitrogenmonoxide; L. masc. suff. -*fex*, the maker; N.L. masc. n. *azooxidifex*, the dinitrogenmonoxide maker (nominative in apposition)].

Basonym: *Rhizobium azooxidifex* Behrendt *et al*. 2016.

The description is as provided by Behrendt *et al*. 2016 [74]. *M. azooxidifex* can be differentiated from other species of the genus *Mycoplana* based on OGRI calculations (ANI and dDDH). The genomic G+C content of the type strain is 64.3 mol%. Its approximate genome size is 5.89 Mbp.

The type strain is DSM 100211^T^ (=Po 20/26^T^=LMG 28788^T^). The NCBI RefSeq assembly accession number for the genome sequence is GCF_014196765.1.

## Supplementary Data

Supplementary material 1

Supplementary material 2
