# Supplementary material for: Taxonomy of Rhizobiaceae revisited: proposal of a new framework for genus delimitation
Source: Int J Syst Evol Microbiol. 2022 Mar 3;72(3):005243. doi: 10.1099/ijsem.0.005243 (PMC9558580; doi:10.1099/ijsem.0.005243)
Supplement: Supplementary material 1 [file ijsem-72-5243-s001.pdf]

Supplementary Material for

Taxonomy of *Rhizobiaceae* revisited: proposal of a new framework for genus delimitation

Nemanja Kuzmanović, Camilla Fagorzi, Alessio Mengoni, Florent Lassalle, George C diCenzo

Florent Lassalle
fl4@sanger.ac.uk

George diCenzo
Email: george.dicenzo@queensu.ca

This PDF file includes:

Figures S1 to S12
Legends for Datasets S1 to S3

Other supplementary materials for this manuscript include the following:

Datasets S1 to S3

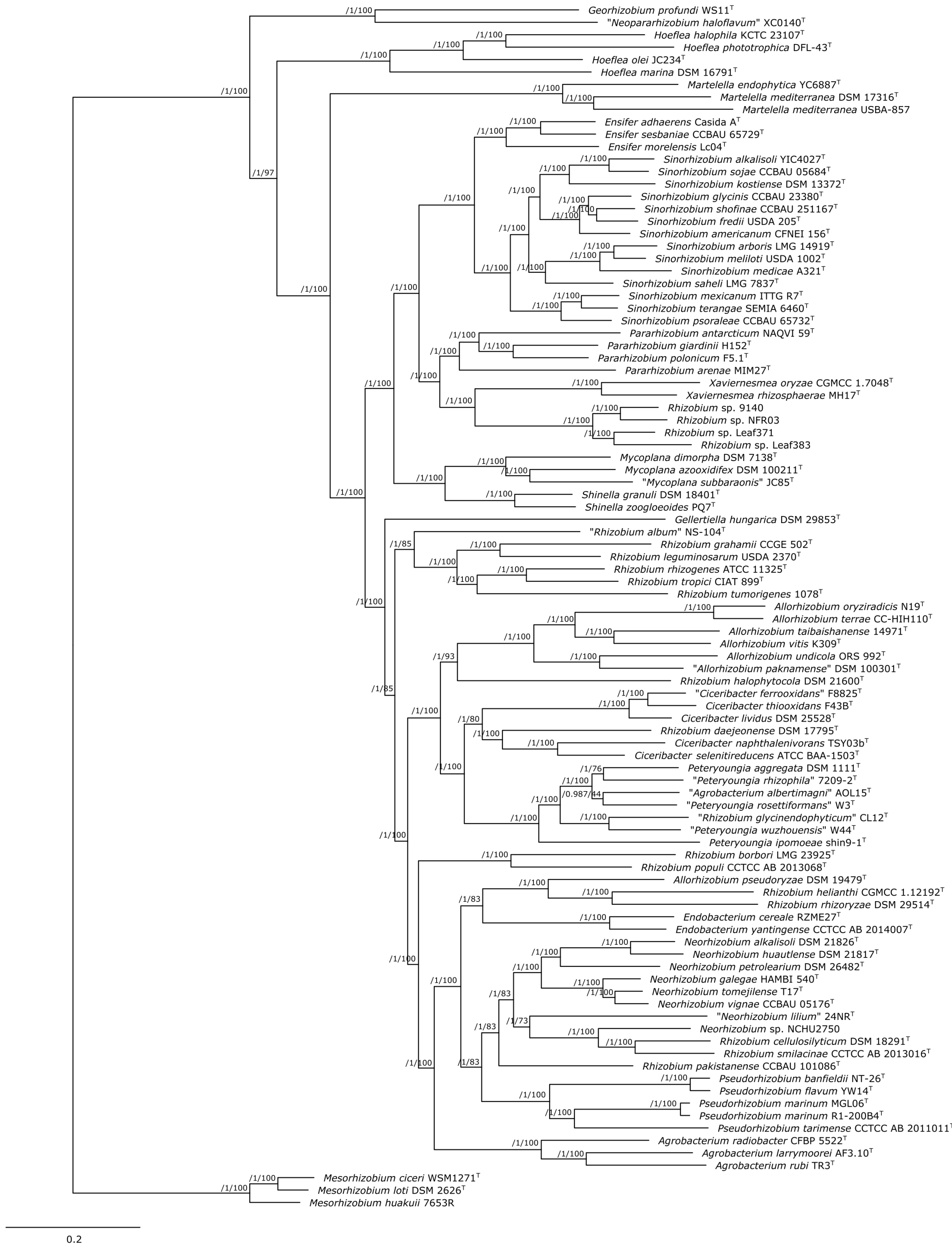

Figure S1. Maximum likelihood core-genome phylogeny of the family *Rhizobiaceae*. A maximum likelihood phylogeny 94 *Rhizobiaceae* strains, and three *Mesorhizobium* spp., is shown. The phylogeny is built from the concatenated alignments of 170 nonrecombinant loci using IQ-TREE. The numbers on the nodes indicate the approximate Bayesian posterior probabilities support values (first value) and ultra-fast bootstrap values (second value). The scale bar represents the number of expected substitutions per site under the best-fitting GTR+F+ASC+R8 model.

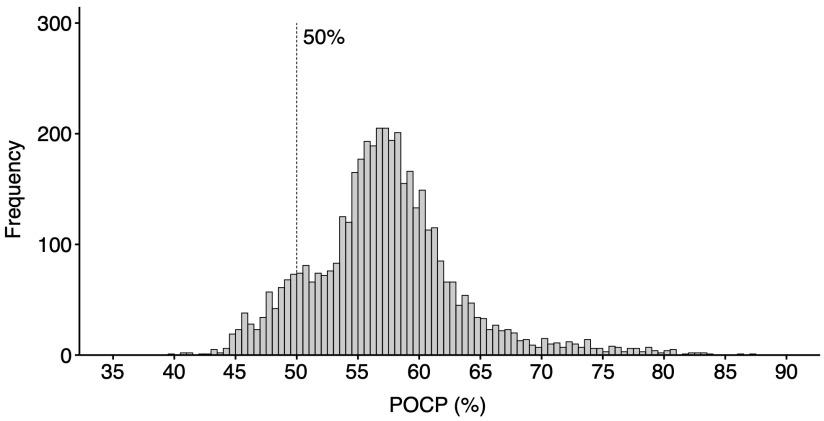

Figure S2. Distribution of percentage of conserved proteins (POCP) comparisons of the family *Rhizobiaceae*. Pairwise POCP values were calculated between 94 members of the family *Rhizobiaceae* as described in the Methods, and the results are summarized as a histogram with a bin width of 0.5%.

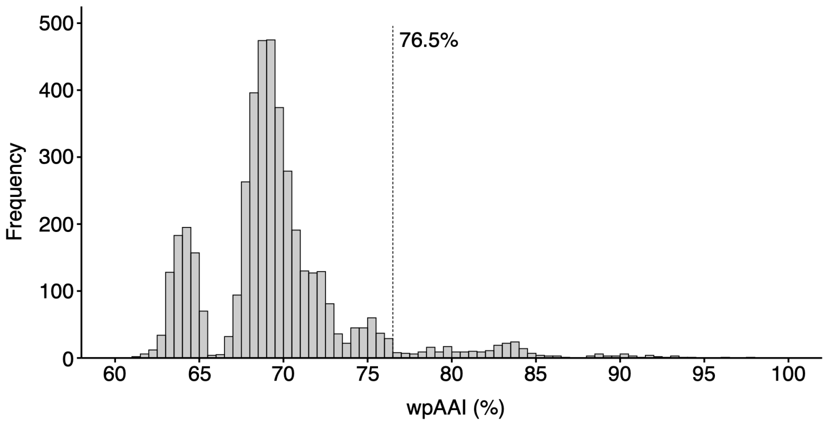

Figure S3. Distribution of whole-proteome AAI (wpAAI) comparisons of the family *Rhizobiaceae*. Pairwise wpAAI values were calculated between 94 members of the family *Rhizobiaceae* using the CompareM workflow, and the results are summarized as a histogram with a bin width of 0.5%.

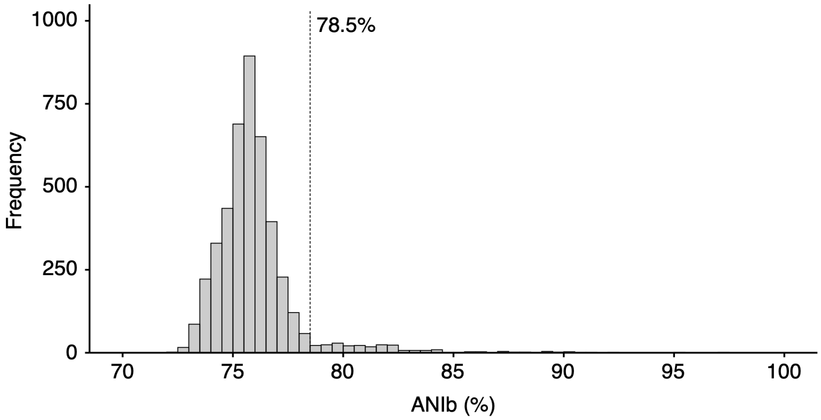

Figure S4. Distribution of average nucleotide identity (ANIb) comparisons of the family *Rhizobiaceae*. Pairwise ANIb values were calculated between 94 members of the family *Rhizobiaceae* using PyANI, and the results are summarized as a histogram with a bin width of 0.5%.

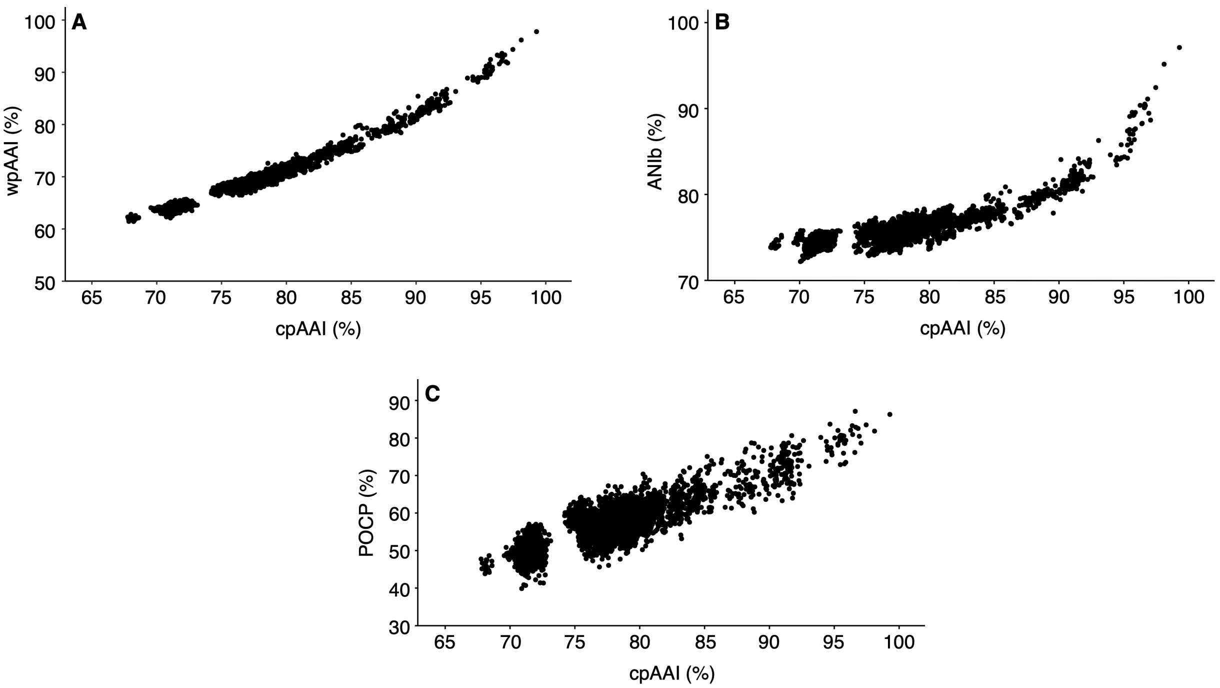

Figure S5. Relationships between OGRIs used in this study. Scatterplots are provided showing the relationships between pairwise cpAAI values and (**A**) pairwise wpAAI values, (**B**) pairwise ANIb values, and (**C**) pairwise POCP values in the family *Rhizobiaceae*.

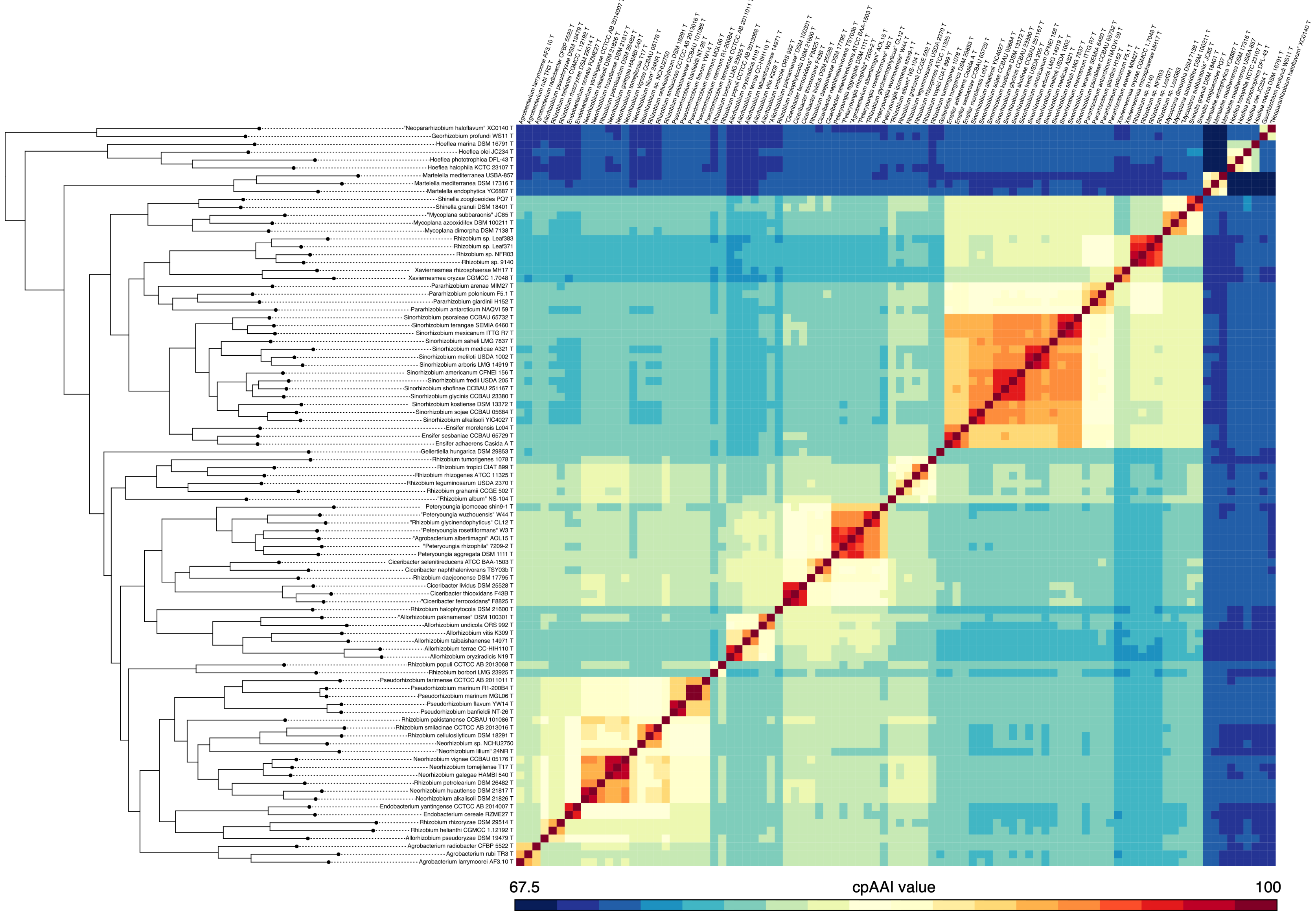

Figure S6. Core-proteome AAI (cpAAI) matrix of the family *Rhizobiaceae*. A matrix showing the pairwise cpAAI values for each pair of 94 members of the family *Rhizobiaceae*. Values were clustered using the core-genome gene phylogeny of Figures 1 and S1.

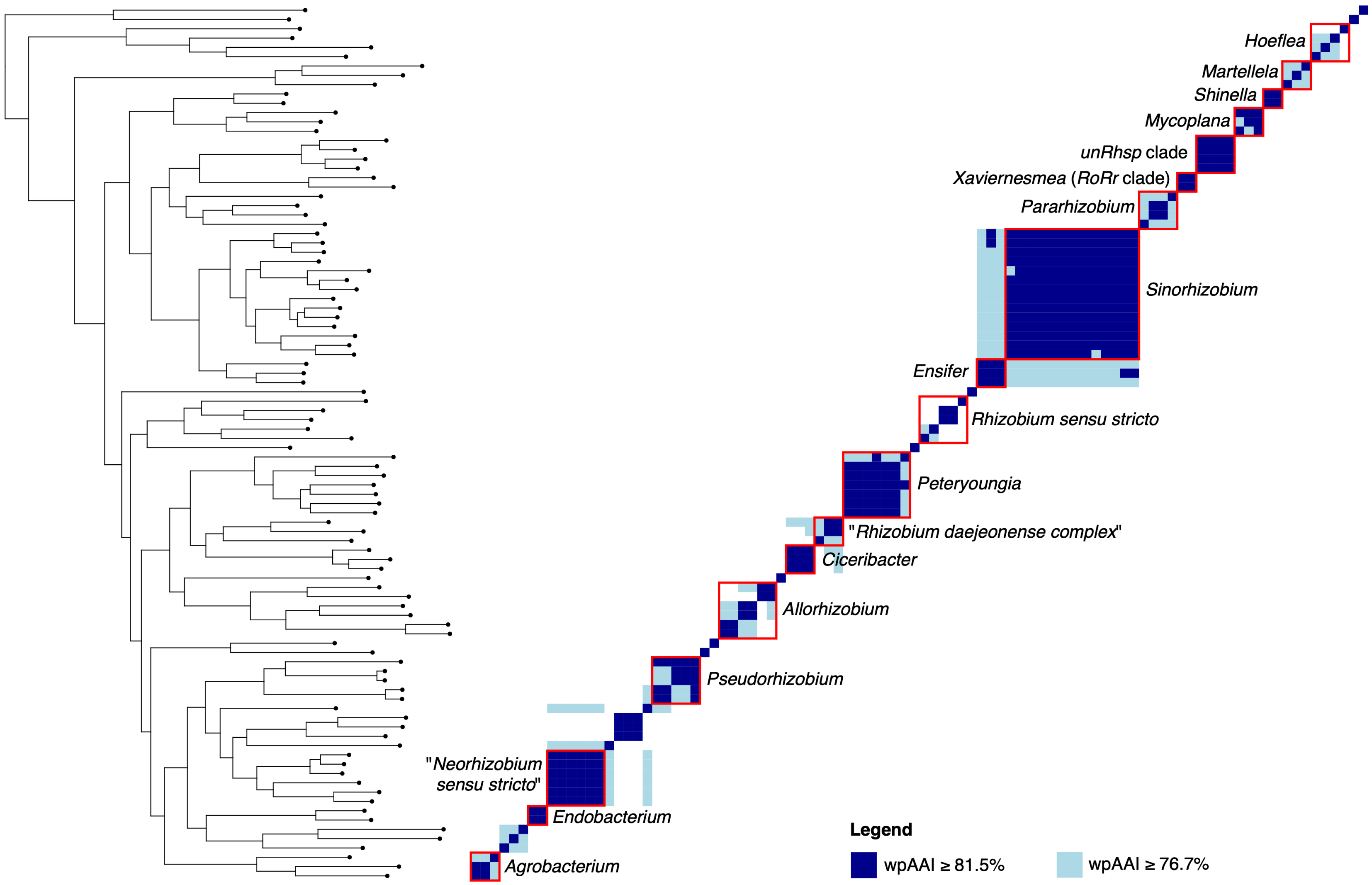

Figure S7. Whole-proteome AAI (wpAAI) matrix of the family *Rhizobiaceae*. A matrix showing the pairwise wpAAI values for each pair of 94 members of the family *Rhizobiaceae*. Values were clustered using the core-genome gene phylogeny of Figures 1 and S1. Several named genera are indicated with red boxes, as indicated. A version of this matrix with a colour scheme representing the full range of cpAAI values is provided as Figure S8.

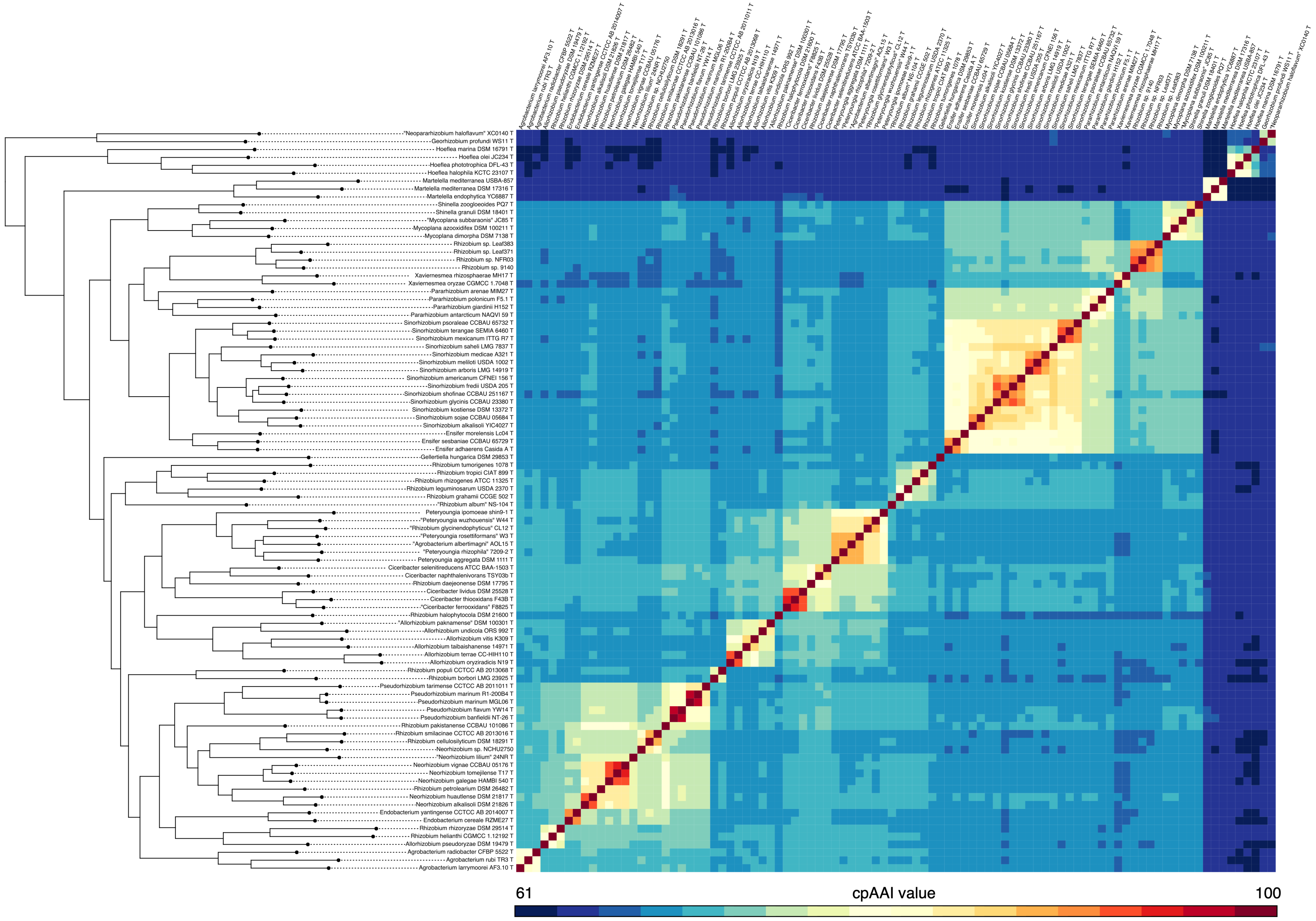

Figure S8. Whole-proteome AAI (wpAAI) matrix of the family *Rhizobiaceae*. A matrix showing the pairwise wpAAI values for each pair of 94 members of the family *Rhizobiaceae*. Values were clustered using the core-genome gene phylogeny of Figures 1 and S1.

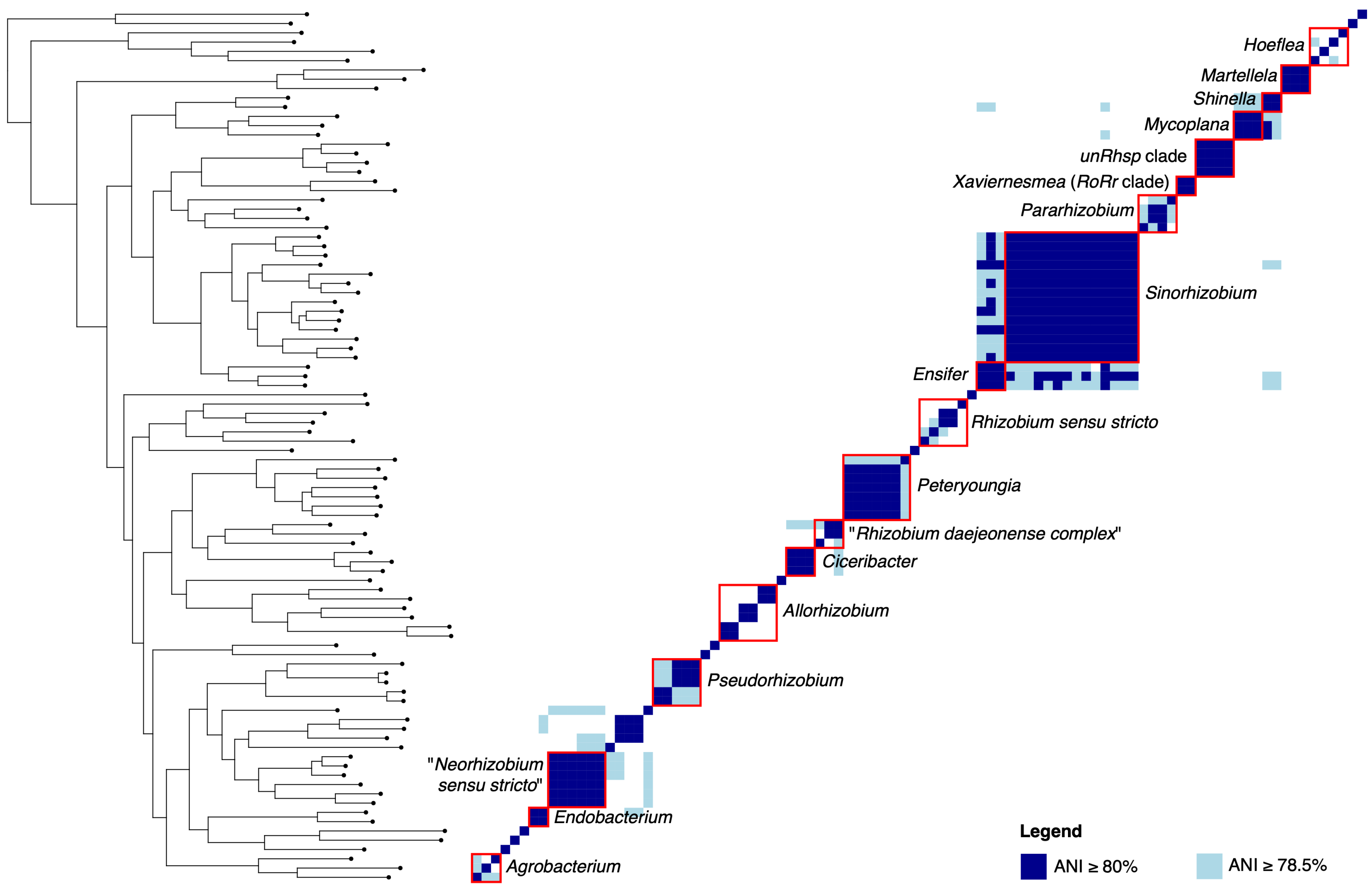

Figure S9. Average nucleotide identity (ANIb) matrix of the family *Rhizobiaceae*. A matrix showing the pairwise ANIb values for each pair of 94 members of the family *Rhizobiaceae*. Values were clustered using the core-genome gene phylogeny of Figures 1 and S1. Several named genera are indicated with red boxes, as indicated. A version of this matrix with a colour scheme representing the full range of cpAAI values is provided as Figure S10.

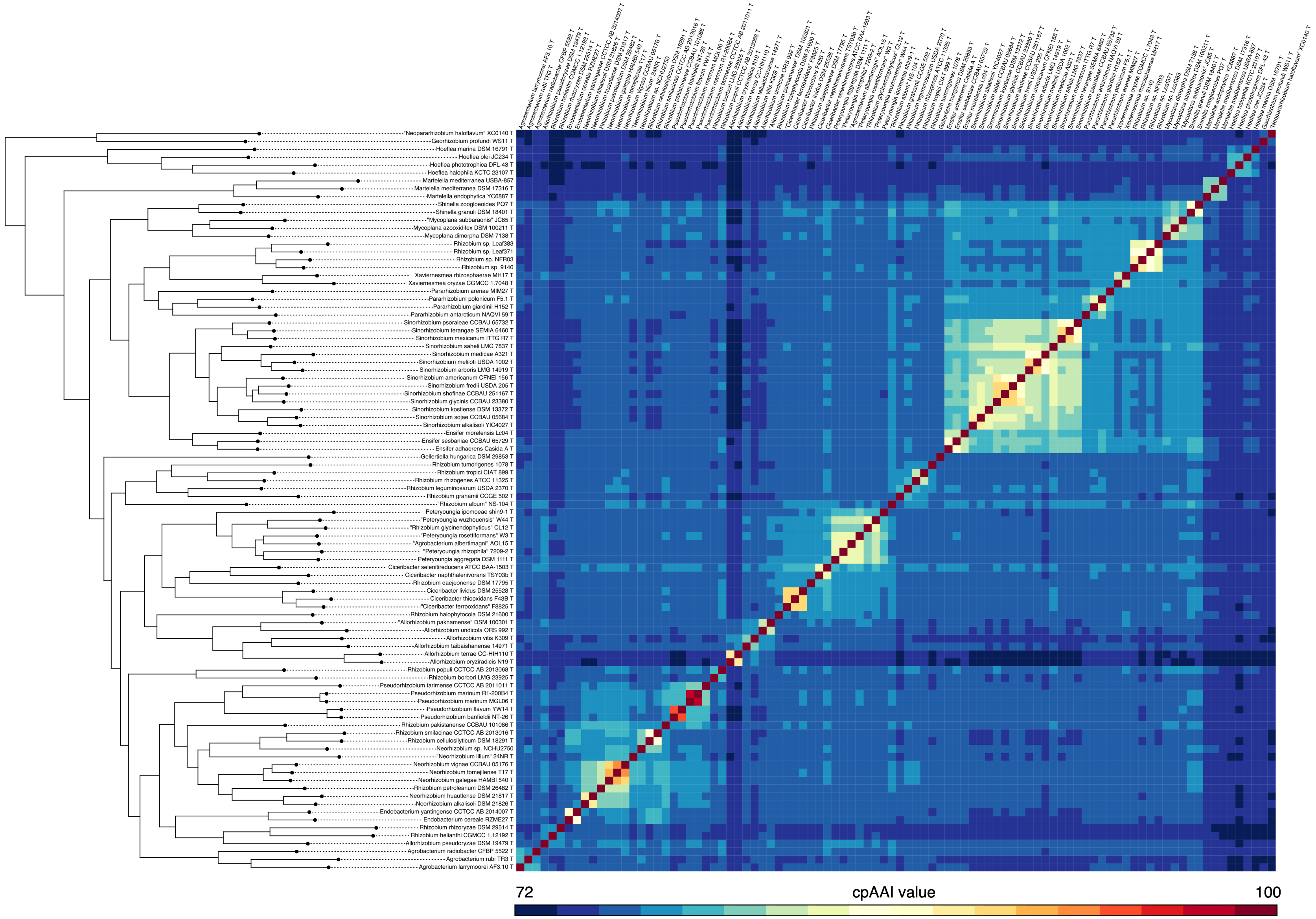

Figure S10. Average nucleotide identity (ANIb) matrix of the family *Rhizobiaceae*. A matrix showing the pairwise ANIb values for each pair of 94 members of the family *Rhizobiaceae*. Values were clustered using the core-genome gene phylogeny of Figures 1 and S1.

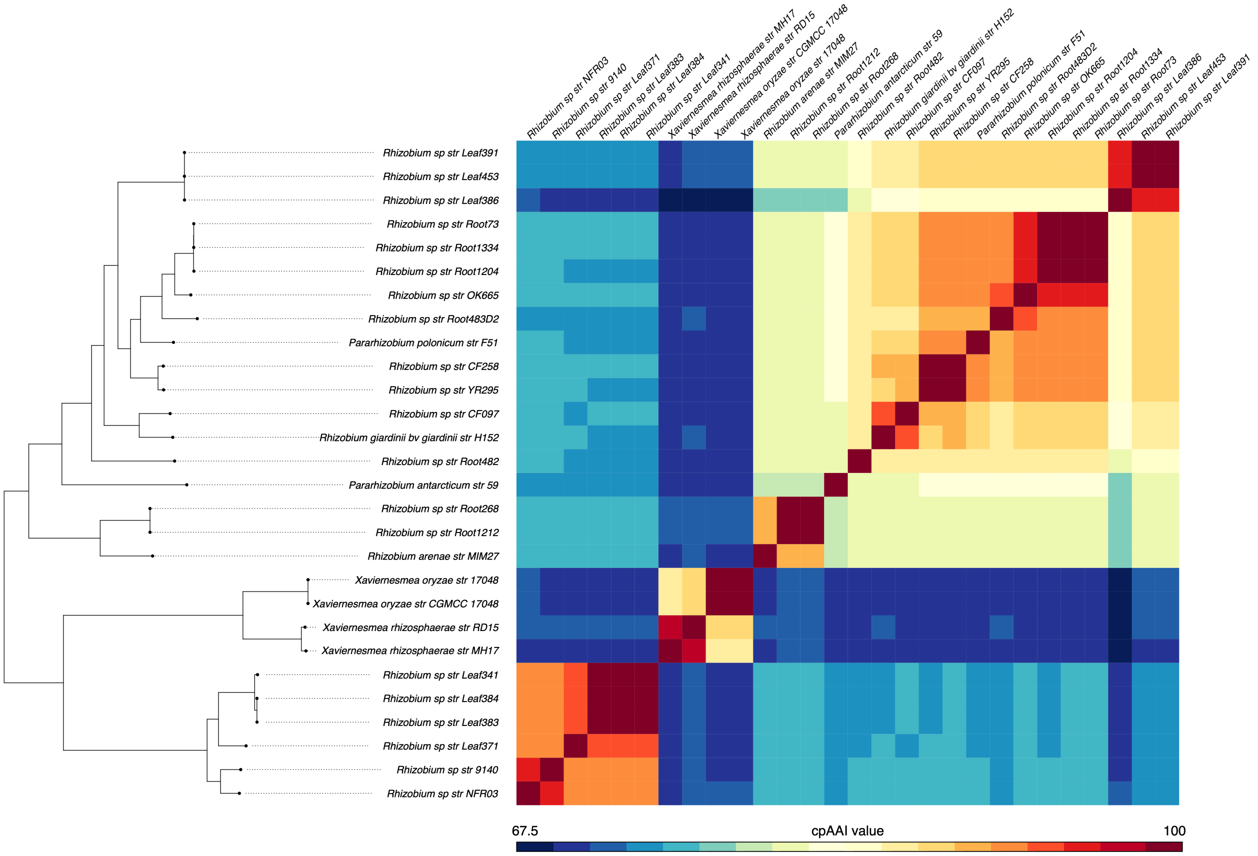

Figure S11. Whole-proteome AAI (wpAAI) matrix of the genus *Xaviernesmea* and related taxa. A matrix showing the pairwise wpAAI values for four members of the genus *Xaviernesmea* together with 24 related organisms.

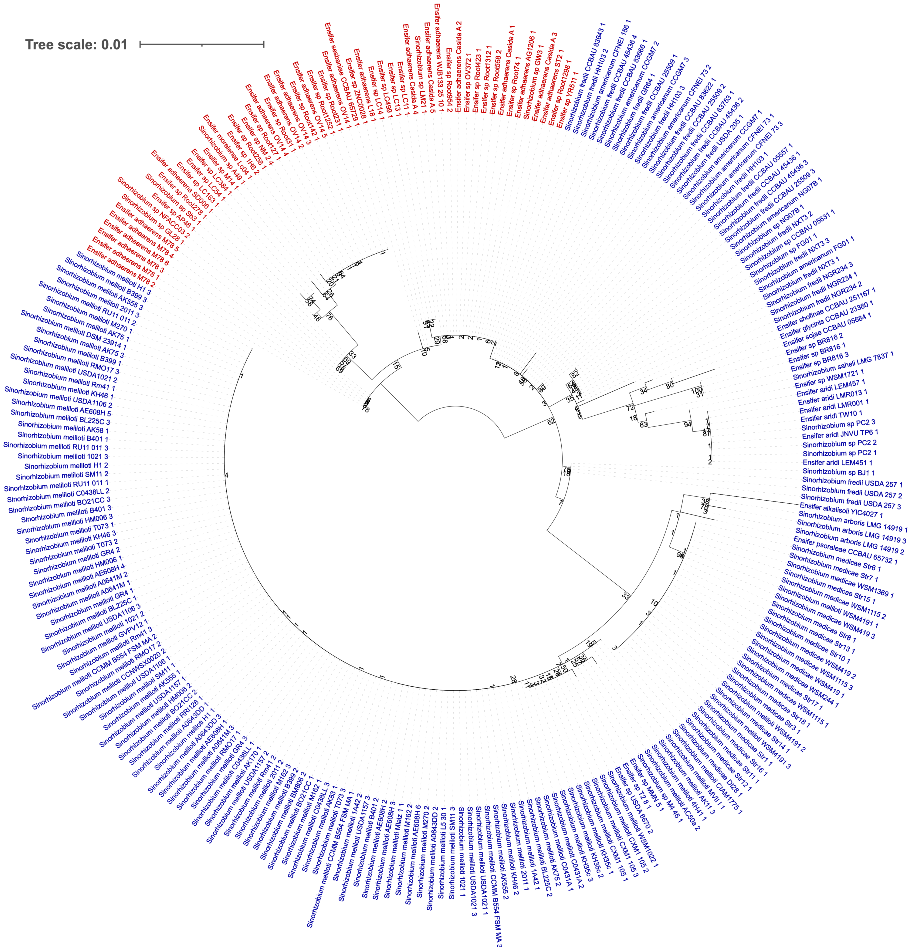

Figure S12. 16S rRNA gene phylogeny of the genera *Ensifer* and *Sinorhizobium*. A maximum likelihood phylogeny of 258 16S rRNA genes from 157 strains of the genera *Ensifer* (red) and *Sinorhizobium* (blue) is shown. Strains are named as recorded in NCBI at the time of collection. The phylogeny was created using RAxML as described in the Materials and Methods. Values represent bootstrap support from 756 bootstrap replicates. The scale represents the mean number of nucleotide substitutions per site. One 16S rRNA gene from each of *E.* sp. GL28 and *S. meliloti* BM806 are not shown due to extremely long branch lengths.

DATASETS

Dataset S1. A list of strains included in our analyses, as well as the corresponding genome assembly accessions.

Dataset S2. Average nucleotide identity (ANIb) values for select taxa from the family *Rhizobiaceae*.

Dataset S3. Digital DNA-DNA hybridization (dDDH) values for select taxa from the family *Rhizobiaceae*.
